# A Comprehensive Review on the Roles of Metals Mediating Insect–Microbial Pathogen Interactions

**DOI:** 10.3390/metabo13070839

**Published:** 2023-07-11

**Authors:** Subhanullah Khan, Minglin Lang

**Affiliations:** 1 CAS Center for Excellence in Biotic Interactions, College of Life Science, University of Chinese Academy of Sciences, Beijing 100049, China; subhani1997edu@mails.ucas.ac.cn; 2 College of Life Science, Agricultural University of Hebei, Baoding 071000, China

**Keywords:** insects, microbial pathogens, metals, physiology, virulence, immune response, acquisition, storage, regulation, toxicity, pest control

## Abstract

Insects and microbial pathogens are ubiquitous and play significant roles in various biological processes, while microbial pathogens are microscopic organisms that can cause diseases in multiple hosts. Insects and microbial pathogens engage in diverse interactions, leveraging each other’s presence. Metals are crucial in shaping these interactions between insects and microbial pathogens. However, metals such as Fe, Cu, Zn, Co, Mo, and Ni are integral to various physiological processes in insects, including immune function and resistance against pathogens. Insects have evolved multiple mechanisms to take up, transport, and regulate metal concentrations to fight against pathogenic microbes and act as a vector to transport microbial pathogens to plants and cause various plant diseases. Hence, it is paramount to inhibit insect–microbe interaction to control pathogen transfer from one plant to another or carry pathogens from other sources. This review aims to succinate the role of metals in the interactions between insects and microbial pathogens. It summarizes the significance of metals in the physiology, immune response, and competition for metals between insects, microbial pathogens, and plants. The scope of this review covers these imperative metals and their acquisition, storage, and regulation mechanisms in insect and microbial pathogens. The paper will discuss various scientific studies and sources, including molecular and biochemical studies and genetic and genomic analysis.

## 1. Introduction

Insects and microbial pathogens carry out many essential biological functions because they are present in varying amounts in nature [1]. Insects are a versatile group of organisms occupying various ecological niches and are requisite for many reasons, including pollination, decomposition, and pest control. However, some insects are pests that pose significant economic and health risks to humans, animals, and plants.

Microbial pathogens, however, are microscopic organisms that can cause disease in various hosts, including insects, humans, plants, and animals [2]. Insects and microbial pathogens interact in a variety of ways. Some insect-specific microbial pathogens have evolved to exploit their insect hosts’ unique physiological and behavioral properties [3]. Other pathogens have a broader host range and can infect multiple species of insects. Insects, in turn, have evolved various mechanisms such as physical barriers, immune responses, and behavioral adaptation to defend themselves against microbial pathogens [4]. The interactions between insects and microbial pathogens involve various physiological and biochemical processes [5]. Metals are a critical factor that plays a crucial role in these interactions. Moreover, the study investigated that metals are pertinent to many physiological processes in insects, plants, and microbial pathogens, including digestion, respiration, and immunity [6]. However, metals can also limit the growth and virulence of microbial pathogens and act as a defense mechanism for insects against infection [7]. Understanding the role of metals in the interactions between insects and microbial pathogens can provide insights into the mechanisms of disease transmission and the development of new strategies to control insect pests and microbial pathogens.

Extensive documentation highlights that metals are essential for various biological processes and important for facilitating interactions among plants, animals, and their environment [8]. Metals involve physiological and biochemical processes, including energy production, enzyme activation, and cellular signaling [9,10]. They also play an indispensable role as a cofactor in many proteins and enzymes, such as hemoglobin, myoglobin, and cytochrome c, involved in respiration and other metabolic pathways [11]. However, various studies have identified that metals play a crucial role in immunological reactions and act as cofactors for enzymes accountable for producing reactive oxygen species (ROS) and other antimicrobial compounds [12]. These metals have toxic effects on living organisms when present in higher amounts. Metals toxicity can disrupt the structure and functions of proteins and enzymes by binding to their functional groups nonspecifically [13]. Toxic metals in higher concentrations produce higher ROS, which has been observed to produce harmful effects on cells and tissues [14]. This review aims to draw together a range of scientific studies and sources, including molecular and biochemical studies and genetic and genomic analysis, on the role of metals mediating insect–microbial pathogen interactions, as well as provide an overview to highlight the importance of metals in biological systems and the potential applications of this knowledge for pest and disease control.

## 2. Role of Metals in Insects

### 2.1. Iron (Fe)

Iron (Fe) plays several vital roles in insects. It is an essential part of hemoglobin and acts as a cofactor in several enzymes involved in various metabolic pathways, such as respiration and energy production [15].

Fe is also crucial for insects’ proper development and growth, as it synthesizes proteins and DNA [16]. The mechanism of Fe absorption in insects encompasses acidification of the midgut, interaction with Fe-binding proteins like transferrin, direct uptake of heme, and storage of excess Fe as ferritin [17]. Acidification of the midgut creates an acidic environment that enhances Fe solubility, allowing for its absorption by gut epithelial cells. Insects also utilize transferrin receptors on gut cells to facilitate the endocytosis of Fe-transferrin complexes [18]. Blood-feeding insects can directly absorb heme from host blood, which is transported across the gut epithelium for various physiological processes. Excess Fe is stored in ferritin, acting as a Fe reservoir that can be utilized when Fe availability is limited. These mechanisms ensure efficient Fe acquisition and utilization in insects for essential physiological functions. It can also improve the insect immune system by producing ROS to inhibit the growth of microbial pathogens [19].

**Figure 1:**
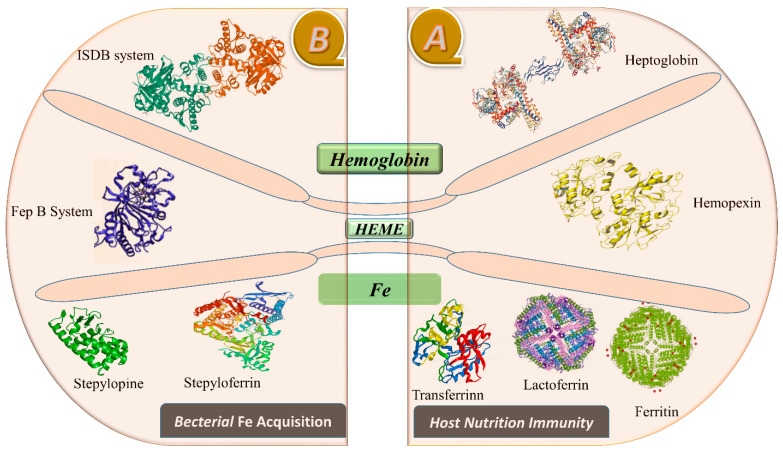
This figure illustrates the proteins recruited in the sequestration of Fe by host nutritional immunity, as shown in (**A**). In contrast, (**B**) depicts the bacterial effectors in Fe retrieval.

Moreover, the study showed that a protein known as ferroportin helps transport and regulate Fe in various types of cells, whether absorbed from the diet via intestinal enterocytes, recycled by macrophages, or stored in hepatocytes. These proteins, such as transferrin, cross the cell membrane to reach the plasma Fe carrier protein [20]. The precise topology and mechanism of Fe transport through ferroportin are not well-understood, and these are considered to be significant unresolved questions in Fe biology. Ferroportin is abundant in specific cells known for Fe export, such as duodenal enterocytes, splenic and hepatic macrophages, and to a lesser extent, hepatocytes [21]. It is also found in the lung, renal tubules, and erythrocyte precursors in the bone marrow [22], although its function in these locations is unclear. Ferroportin transports Fe into the bloodstream on the basal side of enterocytes, and divalent metal transporters expressed on the luminal side of enterocytes regulate Fe absorption in the gut. In the transferrin cycle, the divalent metal transporter actively transfers Fe into enterocytes and is also expressed in macrophages and endosomes [23]. The release of free Fe from the transferrin-receptor complex causes a pH-mediated conformational shift in the endosomes, where it is then transported to the cytoplasm via a divalent metal transporter [24]. Serum transferrin, a member of the transferrin superfamily of proteins, including ovotransferrin and lactoferrin, transports Fe throughout the body. This export protein enables Fe efflux from macrophages and Fe acquisition by enterocytes [25]. Heptaglobin binds hemoglobin to the Heme that is secured by hemopexin. Circulating Hp can effectively manage moderate hemolysis, saturating at 1.5 g/L free Hb [26]. Hepatocytes and macrophages have receptors that uniquely recognize the Hp/Hb complex, as shown in Figure 1.

**Figure 2:**
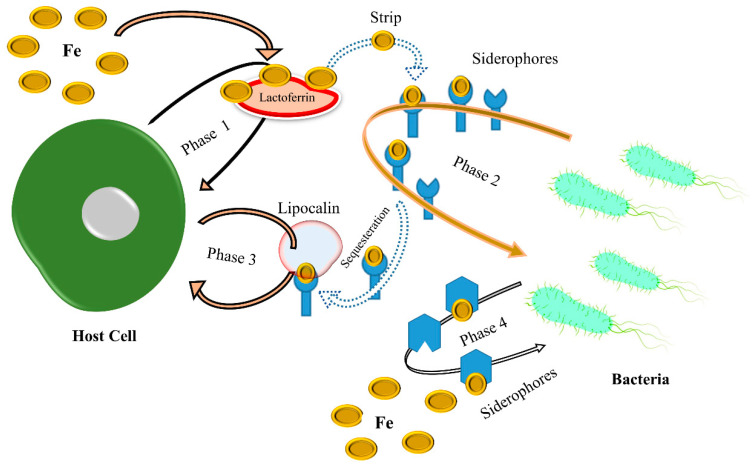
In phase 1, this figure shows that when in a tug-of-war with Fe, host cells produce Fe-binding proteins such as lactoferrin to prevent pathogens from taking up Fe. However, while the pathogens in phase 2 use high-affinity siderophores as a defense mechanism to extract Fe from host proteins, the host cells produce siderophore-binding proteins such as lipocalin to neutralize the siderophores and stop pathogen acquisition, as shown in phase 3. Pathogens can generate siderophores to which lipocalin cannot bind to ensure pathogen survival, as shown in phase 4.

Fe is an essential nutrient for the survival and growth of microbial pathogens, as it is required for several critical physiological processes [27]. However, Fe is not readily available in the host environment, as it is tightly bound to host proteins such as transferrin and lactoferrin to prevent microbial growth [28]. Therefore, microbial pathogens have evolved multiple strategies to harvest Fe from the host. One such strategy is the production of siderophores, small molecules that chelate Fe and facilitate its acquisition from cells [29]. Many bacterial pathogens produce siderophores; some can even intercept siderophores from other microbes. Another strategy is the expression of high-affinity Fe transporters, which actively enable the pathogen to take up Fe from host proteins. However, the Gram-positive pathogen *Staphylococcus aureus* expresses the ScaABC transporter, specific for transferrin-bound Fe [30]. Siderophores are increasingly recognized for their contribution to virulence beyond simple Fe chelation. They also act as signals that elicit a strong host defense, promoting mitophagy, hypoxic responses, and cytokine production [31,32]. Therefore, microbial pathogens must overcome these obstacles to acquire sufficient Fe for survival and virulence. Scrutinizing the mechanisms of Fe acquisition in pathogens is crucial for developing novel antimicrobial strategies [33]. However, targeting siderophores or Fe transporters could limit the growth and virulence of pathogens. For pathogenic bacteria, obtaining sufficient Fe during and after infection inside the host is one of the main barriers. At the host–pathogen interface, an analytical structure describes the flow of signaling and the struggle for shared resources between the host and pathogen, and host–pathogen competition for this valuable transition metal take place, as shown in Figure 2. In addition, Fe-based therapies, such as Fe chelation therapy, have also been studied as potential treatments for infectious diseases [34]. However, the efficacy of such therapies has yet to be determined, as Fe is integral to host physiology and immune function.

It has been widely recognized that microbial pathogens depend on acquiring and utilizing Fe from their host for survival. However, the host immune system’s mechanisms for retaining Fe limit this process [35]. Several Fe acquisition mechanisms have evolved in microbial pathogens to overcome this limitation, including the production of siderophores, Fe transporters, heme acquisition systems, and Fe-regulated surface proteins. Siderophores are small Fe-chelating molecules secreted by pathogens that bind and transport Fe into the cell [36]. Fe transporters and heme acquisition systems help pathogens acquire Fe from host proteins, while Fe-regulated surface proteins capture Fe from host transferrin and lactoferrin. Fe-responsive regulators such as Fur tightly regulate Fe acquisition in pathogens, which regulate the expression of genes involved in Fe acquisition and metabolism [37,38]. Fe acquisition mechanisms such as siderophores and Fe transporters in bacterial infections are crucial virulence factors that allow bacteria to overcome host Fe retention mechanisms and establish conditions [39]. Fe acquisition mechanisms such as high-affinity Fe transporters and Fe-responsive regulators are pertinent to fungal virulence and pathogenicity in fungal infections. In parasitic infections, Fe is involved in several essential processes, such as energy production, DNA synthesis, and oxidative metabolism, and is prominent for the parasite’s survival and growth in the host [40]. The role of Fe in interactions between insects and microbial pathogens is complex and diverse, with both essentiality and toxicity playing integral parts.

### 2.2. Interaction of Plants with Insects and Microbes via Fe

There is substantial proof that Fe is an essential nutrient for plants and many microbes and necessary for their growth and development. The availability of Fe can influence plant–insect and plant–microbe interactions [41], and Fe-rich soils can increase the abundance and activity of certain beneficial microbial species, such as mycorrhizal fungi. These rhizobacteria can help plants become more herbivore-tolerant and improve their defenses against herbivores [42,43]. Moreover, these beneficial microbial species may also prevent and reduce the populations of certain insect pests [44]. In addition, certain insect species can give plants access to Fe-rich food sources of nutrition by bringing Fe-rich soil particles to the root zone as shown in Table 1. However, Fe-deficient soils can negatively impact plant-microbial interactions, and specific plant pathogens can exploit the lack of Fe to infect and damage plants [45].

The plant Fe deficiency response is regulated at the transcriptional and post-translational levels. Hormones like auxin, ethylene, nitric oxide, cytokinin, and gibberellic acid play vital roles in this process [46]. In Arabidopsis, ethylene and gibberellic acid enhance Fe uptake by increasing FRO2 and IRT1 expression. Ethylene and auxin promote nitric oxide accumulation, stabilizing FIT and improving Fe uptake. Auxin also stimulates lateral root formation for increased Fe absorption [47].

Conversely, cytokinin inhibits root growth and suppresses Fe deficiency response genes: salicylic acid and jasmonic acid, two major defense hormones, influence plant Fe acquisition [48]. Salicylic acid positively affects Fe uptake gene expression in Arabidopsis through auxin and ethylene signaling [49]. On the other hand, jasmonic acid negatively regulates Fe acquisition by downregulating Fe uptake genes independently of FIT [50]. Ethylene and auxin hormones are crucial in the plant immune signaling network. This connection between Fe availability and immunity highlights their potential role in Fe uptake responses in plant roots.

### 2.3. Zinc (Zn) and Copper (Cu)

Like humans, insects rely on dietary intake of trace metals like Zn and Cu for proper physiological functioning. These specified metals are responsible for multiform insect processes, including DNA synthesis, oxidation reactions, cuticle biosynthesis, and acting as essential cofactors for numerous enzymes [63]. Their presence is indispensable for insects’ everyday functioning and overall well-being at the molecular and biochemical levels. In insect studies, two families of Zn transporters: ten dZip and seven dZnT proteins, analogous to human Zip (SLC39) and ZnT (SLC30) families [64]. These transporters play roles in Zn influx and efflux, with specific expression in the midgut (dZip1) and Malpighian tubules (dZnT35C), contributing to Zn absorption and excretion. Zn is distributed throughout the gastrointestinal tract, with higher accumulation in the posterior midgut, crop, and Malpighian tubules [65]. dZip1 and dZip2 import Zn from the lumen into the enterocyte, while dZnt1 and ZnT77C release imported Zn into circulation from the basolateral membrane. The silencing of dZnt1, specifically in the gut, increases lethality under Zn-deficient conditions, highlighting its crucial role in Zn absorption [66]. Zn repletion leads to the suppression of dZip1 and dZip2 mRNA expression and the protein expression of dZnt1 [67].

The expression of dZnt1 and dZnT35C, a potential ZnT2 homolog, is regulated by dMTF1. FOI, an ortholog of dZip6 and dZip10, is essential for cell migration and gonad morphogenesis by controlling DE-cadherin expression at the posttranscriptional level [66]. A Catsup mutant with a defective dZip7 exhibits high levels of catecholamines and shows signs of semi-dominant lethality. The mutant also displays defects in membrane protein trafficking and increased ER stress [68]. On the other hand, Zn is embarked on regulating gene expression in insects, where it acts as a cofactor for several transcription factors that control gene expression [69]. Zn is also involved in regulating insect development, which plays a crucial role in insect cuticle formation and the regulation of molting.

The most prominent finding to emerge is that Cu is a crucial part of the innate immune system of insects, where it acts as a cofactor for the enzyme phenoloxidase. Phenoloxidase plays a significant role in the insect immune response by catalyzing the oxidation of phenolic compounds to quinones, which are toxic to microorganisms [70,71]. The quinones also contribute to the formation of melanin, which is pivotal for encapsulating pathogens via insect defense cells.

Phenol + O_2_ + Cu^2+^ → Quinone + H_2_O + Cu*^+^*

Quinone + Quinone + Cu^+^ → Melanin + Cu^2*+*^

In this reaction, the copper becomes an ion (Cu^2+^), acting as a cofactor for the phenoloxidase enzyme and facilitating the transfer of electrons during the oxidation of phenols. The reaction cannot proceed without the Cu ion, and melanization cannot occur, making the insect vulnerable to foreign invaders.

Furthermore, Cu also regulates the insect’s antioxidant defense system, which protects the insect’s cells from oxidative damage caused by the ROS produced during the immune response [72]. However, it has been reported that dMTF-1 is a crucial regulator of essential metal homeostasis, controlling gene expression in metal pathways [73]. DmATP7 is vital for Cu uptake and efflux in insects, particularly during larval development. DmATP7 term depends on functional dMTF-1, while its background expression is maintained in dMTF-1 knockout flies [74]. dMTF-1 also regulates the Cu importer protein Ctr1B in response to Cu-specific stress, facilitating increased Cu uptake. Knockout flies lacking dMTF-1 exhibit decreased survival and prolonged development due to impaired metal regulation [75]. dMTF-1 is also crucial for transcription factors; an insect tightly regulates these metals. It controls the expression of ZnT and Zn exporter proteins involved in the uptake and efflux of Zn while also regulating Cu-related genes. dMTF-1’s concentration gradient between the cytosol and nucleus governs the regulation of Zn exporters [76]. Zn toxicity induces dMTF-1 upregulation and translocation into the nucleus, where it binds to the MRE upstream of ZnT, promoting ZnT transcription and Zn exporter production [77]. Although the regulation of Zn importers via dMTF-1 in insects is not yet established, it is possible that some Zn importers may also be influenced by dMTF-1. This regulatory mechanism involving dMTF-1 ensures the maintenance of Zn and Cu homeostasis, essential for RNA and DNA metabolism. On the other hand, Zn is required for the proper functioning of several immune-related enzymes, including alkaline phosphatase and carbonic anhydrase. These enzymes play an integral role in the insect’s immune response by regulating the insect’s tissue pH and modulating the immune cells’ activity [78]. Zn is also immersed in regulating the expression of several immune-related genes in insects, including genes encoding antimicrobial peptides, which are essential in the insect’s defense against microbial pathogens [79].

A number of studies have disclosed that Zn and Cu are crucial cofactors for various enzymes involved in multiple biochemical reactions in insects. Zn is required to function in enzymes integrated into DNA synthesis, RNA transcription, and protein synthesis, as well as repair enzymes properly [80]. Zn ions (Zn^2+^) are coordinated to the amino acid residues of DNA polymerases, such as DNA polymerase III. This coordination stabilizes the enzyme’s binding to the DNA template and allows for the accurate replication of genetic information [81,82]. Zn ions interact with the negatively charged phosphate backbone of the DNA molecule and form coordination complexes that stabilize the enzyme-DNA complex. This coordination also helps to properly position the deoxynucleoside triphosphate (dNTP) substrates for incorporation into the growing DNA chain, resulting in the actual complexity of the genetic information [83]. In addition to its role in DNA polymerase activity, Zn is also involved in the movement of other enzymes involved in DNA synthesis and repair, such as DNA ligases and topoisomerases. Zn ions cooperate in binding these enzymes to DNA substrates, allowing for the efficient repair of DNA strand breaks and the accurate replication of genetic information [84].

**Figure 3:**
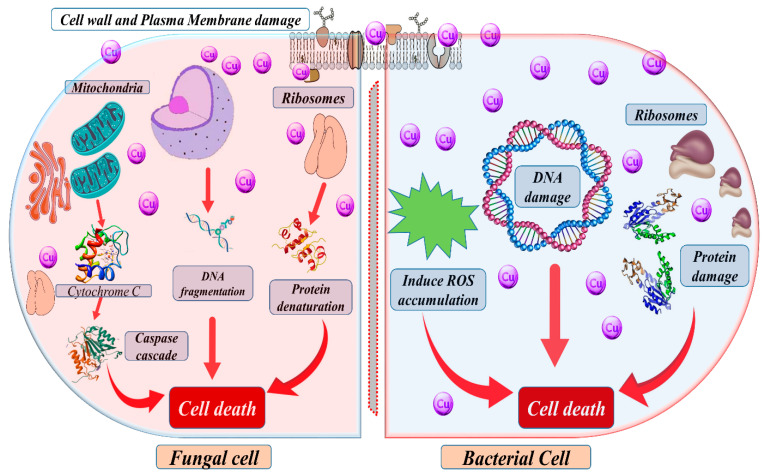
A schematic diagram depicting Cu’s potential mechanism of action on microorganisms, which can be proposed as follows: Cu impacts the cell wall of microbes, disrupting its constituents and damaging the membrane. This membrane damage subsequently reduces the electrochemical potential, compromising the integrity of the membrane. Furthermore, Cu specifically targets microorganisms’ DNA, interfering with the synthesis of proteins and inducing detrimental effects that eventually lead to the demise of the microbial cell.

Moreover, the host immune system tightly regulates the availability of these metals in the host environment, which can limit their accessibility to invading pathogens. In microbial pathogens, Zn can also control the expression of virulence factors and the formation of biofilms, which can enhance their ability to colonize and infect the host [85]. However, the host immune system can absorb Zn through various mechanisms, such as the production of metal-binding proteins, to limit its availability to invading pathogens [86]. Cu is an essential cofactor for many enzymes involved in cellular processes, including respiration, response to oxidative stress, and Fe acquisition, as well as control the virulence factor and biofilm formation in pathogens. However, excess Cu can be toxic to cells by generating ROS, which can damage DNA, proteins, and lipids [87], as shown in Figure 3. Hence, microbial pathogens have evolved various mechanisms to deal with excess Cu, such as producing Cu-binding proteins and activating detoxification systems. Overall, the roles of Zn and Cu in microbial pathogens are complex and tightly regulated by the host immune system [86].

### 2.4. Interaction of Plants with Insects and Microbes through Zn and Cu Metals

Plants interact with insects and microbes by competing with them to gain metals such as Zn and Cu. Within the realm of enzyme activity, Zn is essential for the proper function of DNA/RNA polymerase enzymes, ribosomes, and superoxide dismutase (SOD) [88]. It exhibits specific significance in plants, present in carbonic anhydrase and stromal processing peptides, thereby contributing to photosynthesis [89]. Furthermore, Zn contributes to protein structure, with approximately 4% of Arabidopsis proteins containing Zn finger domains, emphasizing its functional importance in plant physiology [90]. Cu is imperative for active functioning critical enzymes such as cytochrome oxidases, ascorbate oxidase, superoxide dismutase (SOD), and polyphenol oxidase [91,92]. In plants, Cu is also required for the receptor signaling of the hormone ethylene, which plays a crucial role in plant development and disease resistance [93]. These metals are needed to form chlorophyll, which is pivotal for photosynthesis, and for auxin production. This hormone stimulates cell division, elongation, tissue differentiation, and tropism (responses to environmental stimuli) [94]. In addition, these metals can be used by insects and microbes as energy sources and for the biotransformation of compounds, such as nitrogen and sulfur. In addition, Zn and Cu can be used by plants to ward off herbivores, as they are toxic to certain insects [95]. However, Zn toxicity occurs in agricultural soils treated with sewage sludge, in urban and suburban soils enhanced via anthropogenic inputs of Zn, especially in soils with low pH, and in soils affected by mining and smelting activities [96]. Mechanisms for creating either low or high Zn scenarios in plant and animal systems are essential for Zn-based disease and pest control.

Eukaryotic cells have an impressive ability to regulate the levels of Zn within their interiors. Despite Zn being commonly present in lower concentrations at the interiors of the cell, it is present in higher ranges outside the eukaryotic cells [97]. A diverse range of proteins, such as ZIP (ZRT- and IRT-like proteins), ZNT (Zn transporter), and metallothioneins (MTs) that sequester Zn, are involved in regulating Zn equilibrium in plants. ZNT proteins transport Zn within cells, averting cytotoxicity by sequestering it within vacuoles, while ZIP proteins facilitate the absorption of Zn from the soil into plant root cells [98]. MTs act as Zn chelators, storing excess Zn in a harmless form, protecting against Zn deficiency. This cooperative system ensures Zn’s controlled distribution, storage, and acquisition. ZIP transporters respond to low Zn levels by upregulation, whereas ZNT transporters and MTs become active when Zn levels are high, guaranteeing proper physiological development and plant function [14,99].

**Figure 4:**
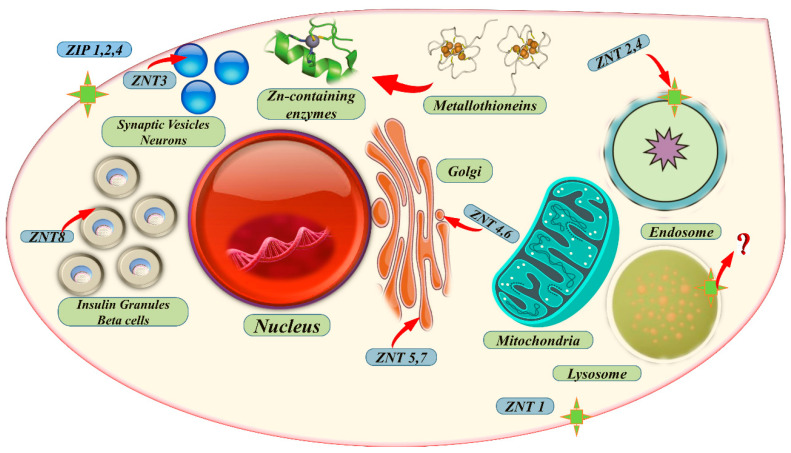
The localization and transport of Zn within crucial cells. This figure illustrates the processes involved in ZIP and ZNT Zn transporter families. The arrows indicate the movement of Zn. When Zn levels are low, ZIP1, ZIP2, and ZIP4 transporters become active, while Zn administration stimulates the expression of ZNT-1 and ZNT-2 transporters. Typically, a higher Zn efflux increases susceptibility to apoptosis, whereas elevated Zn levels offer protection and promote autophagy.

In contrast, ZNT proteins decrease intracellular Zn levels by promoting Zn release from the cell or its uptake into intracellular vesicles. The sequestration of Zn is primarily controlled by Zn-dependent mechanisms that regulate the transcription, translation, and intracellular trafficking of these transporters [100]. Indeed, many studies show that the expression levels of Zn transporters in plant tumors have been found to correlate with the severity of malignancy, indicating that disruptions in intracellular Zn homeostasis can contribute to cancer progression. In various types of cancer, specific Zn importers are upregulated, potentially enabling tumor cells to evade programmed cell death (apoptosis) and activate survival mechanisms through autophagy [101,102]. ZIPs and ZNTs are among the critical Zn transporters involved in these processes, as shown in Figure 4. In addition, microbes can use these metals to make antibiotics that plants can use to protect against infection [103].

Zn and Cu can be found in soil and taken up by plants, and are also present in the bodies of insects and microbes [104]. Zn also helps protect plants from diseases caused by fungi, bacteria, and viruses by producing phytohormones, such as salicylic acid, and activating the plant’s defense mechanisms [105]. Zn can also induce the expression of defense-related genes, such as those involved in synthesizing phytoalexins, which are antimicrobial compounds that may help plants ward off disease [106]. In addition, Zn can stimulate the production of secondary metabolites, such as flavonoids, with antimicrobial properties [107,108]. Zn also helps maintain the structural integrity of plant tissues, which can prevent pathogen invasion.

Cu is significant for enzymes to function properly and to protect plants from environmental factors such as cold, heat, and drought [109]. One of the main functions of Cu in plants is as a cofactor for enzymes implicated in various metabolic pathways, including photosynthesis, respiration, and lignin synthesis. It serves as a component of the primary electron donor in the photosystem 1 of plants [110]. Due to its ability to readily gain and lose electrons, Cu acts as a cofactor for oxidase, mono, and di-oxygenase enzymes such as amine oxidases, ammonia monooxidase, ceruloplasmin, and lysyl oxidase. Additionally, Cu is involved in the function of enzymes responsible for eliminating superoxide radicals, including superoxide dismutase and ascorbate oxidase [111]. It can also improve plants’ resistance to these stresses by increasing antioxidant activity and reducing oxidative damage. Additionally, Cu is pertinent in maintaining membrane integrity and stability, which can help prevent water loss during drought and cold stress [112].

Insects and microbes are vital to plants’ health, as they help to provide plants with essential nutrients and water [113]. Microbes also help break down soil nutrients, making them available to plants. Insects can also provide prominent nutrients to the plant, such as nitrogen, phosphorus, and potassium, and other beneficial compounds such as Ca, Mg, and S, as well as the vitamins and hormones necessary for the proper functioning of enzymes and other metabolic processes in plants [114,115]. In addition to providing essential nutrients, insects can transfer other beneficial compounds to plants. However, some insects can transmit plant-growth-promoting hormones such as gibberellins and auxins to stimulate plant growth and development [116]. Insects can also transfer vitamins and antioxidants such as vitamins C and E, which may help protect plants from oxidative damage [117].

There is substantial proof that Zn and Cu can benefit insects and microbes. Zn is essential for the growth and development of insects and can also help protect them from disease (Table 2). Other metals like Cu are also imperative for the functioning of enzymes in insects and help protect them from environmental stressors [118]. Zn and Cu are paramount for the health of plant-growth-promoting rhizobacteria as they help break down organic matter and make nutrients available to plants. It activates defense mechanisms, induces expression of defense genes, increases the activity of enzymes involved in ROS production, stimulates the production of secondary metabolites, and helps maintain the structural integrity of plant tissues [119]. Therefore, to probe the mechanisms by which metals increases plant disease resistance can help us to develop sustainable crop protection and production strategies in the face of changing environmental conditions.

### 2.5. Metals Other Than Fe, Cu, and Zn

Metals other than Fe, Cu, and Zn, like manganese (Mn), nickel (Ni), cobalt (Co), and molybdenum (Mo), are also essential trace elements that play prominent roles in the growth and development of plants, insects, and microbes [128]. Metal ions, mainly through the Haber–Weiss reaction, are pivotal for oxidative modifications of free amino acids and proteins [129]. Commonly oxidized amino acid residues include histidine, arginine, lysine, proline, methionine, and cysteine. These site-specific modifications occur at metal binding sites within proteins [130]. One significant consequence of oxygen-free radical-induced protein damage is their susceptibility to protease degradation [131].

Additionally, protein oxidation can release its binding metals, such as Fe^2+^ from [4Fe-4S] clusters found in certain dehydratases like aconitases [132]. On the other hand, metal (e.g. Mn, Ni, Co, etc.) binding to the cell nucleus leads to genotoxic damage, including DNA base modifications, DNA–protein cross-linkages, DNA strand breaks, rearrangements, and depurination [133]. Reactive oxygen species generated via metal-mediated production induce pro-mutagenic adducts, such as 8-oxoG (8-oxo guanine), which can cause C to T transversion mutations without DNA repair [134]. Metal-induced carcinogenicity and acute toxicity involve oxidative damage, DNA methylation aberration, and chromatin condensation [135].

Manganese (Mn) is involved in synthesizing chitin, a component of the insect’s exoskeleton. In addition, it also plays a significant role in the development of reproductive organs and is necessary for the appropriate maturation of eggs in certain insect species [136]. Ni is implicated in the metabolism of carbohydrates, amino acids, and lipids and is necessary for synthesizing enzymes that are pivotal for insect growth and development [137]. Co is essential for the metabolism of carbohydrates, amino acids, and lipids and is involved in the synthesis of hemoglobin, which is vital for insect oxygen transport [138]. However, Mo is an essential nutrient utilized as a prosthetic group in oxidoreductases. Its molybdoenzymes, identified in Drosophila, play crucial roles in metabolism, including the breakdown of acetaldehyde and purines [139].

Furthermore, these metals also play a pivotal role in the physiology and pathogenesis of the microbial pathogens, in which delicate mechanisms have evolved to acquire and regulate their levels from the host environment [140]. Mn is vital for the growth and survival of bacterial pathogens and contributes to biological processes like oxidative stress management and DNA protection [141]. Mn is also enlisted in expressing virulence factors such as adhesins and capsules in bacterial pathogens [142]. Moreover, it can affect the stability and folding of proteins involved in the virulence factor synthesis, such as capsule polysaccharides [143]. Subsequently, Ni is considered the most critical cofactor for several enzymes involved in energy metabolism and nitrogen fixation and is required for the growth and survival of many bacterial pathogens. Bacterial pathogens have undergone evolutionary adaptations in their acquisition mechanisms, enabling them to effectively regulate nickel (Ni) levels from the host environment [144]. Ni is also a critical component of some virulence factors such as urease in bacterial pathogens [145]. Co is a component of vitamin B12, which is pivotal for the growth and survival of many bacterial pathogens [146,147], while it can also regulate virulence factors, such as siderophores, in the bacterial pathogens [86]. Moreover, the bacterial virulence factors such as adhesins and capsules also require an optimum concentration of Mo for their expression [148], and Mo is also an essential cofactor for the function of various enzymes involved in redox reactions, including nitrate reductase, formate dehydrogenase, and aldehyde oxidase [149].

2 Mo(VI) + 3 NADH + 9 H^+^ + 2 NO_3_^−^ → 2 Mo(IV) + 3 NAD^+^ + 6 H_2_O + 2 NO_2_^−^

Mo(VI) + NADH + H^+^ + HCOOH → Mo(IV) + NAD^+^ + H_2_O + CO_2_

Mo(VI) + H_2_O + RCHO → Mo(IV) + 2 H^+^ + RCOOH

In redox reactions, Mo acts as a catalytic virtuoso, facilitating the transfer of electrons and protons between the substrates, esteemed cofactors (such as NADH), and the ultimate products. The presence of Mo is crucial for the proper functioning of these enzymes and their involvement in redox processes.

## 3. Role of Metals in Microbial Physiology and Virulence

Trace metals are crucial for developing physiology and virulence in microbial pathogens, as they rely on these metals to facilitate essential biological processes and augment their pathogenic potential. For example, Zn is required for the fungal pathogen *Candida albicans* to express virulence factors such as adhesins and invasins. Moreover, its acquisition mechanisms have also evolved in *C. albicans*, allowing the fungus to acquire Zn from the host environment through manipulating proteins like Zn transporters and Zn-regulated transcription factors [150]. Cu is paramount for several biological processes in microbial pathogens, such as oxidative stress resistance, energy metabolism, and DNA synthesis, for example, the bacterial pathogen *Pseudomonas aeruginosa* requires Cu for siderophores and exotoxin expressions [151]. *P. aeruginosa* can also acquire Cu by producing Cu transporters and Cu-regulated transcription factors [152].

Furthermore, in *Streptococcus pneumoniae,* Mn has been identified as necessary to grow and produce a cytolytic toxin known as pneumolysin, which facilitates pneumococcal infections [153,154]. Besides pneumolysin production, Mn has a significant aspect in gene regulation in responsible for stress resistance and DNA protection in this microbe. While in *S. aureus*, both adhesins expression and biofilm formation, which carries out energy metabolism and oxidative stress resistance, are required to develop an infection in the host [155], where Mn is needed for gene regulation [156]. Developing these virulence factors, such as adhesins, capsules, and other surface-associated proteins, generally helps in the bacterial invasion, colonization, and evasion from the host immune responses [154].

Subsequently, copious bacterial pathogens like *Helicobacter pylori* and *Klebsiella pneumonia* required Ni for their growth and physiological development. For example, in these bacteria, Ni acts as a cofactor in urease enzymes to stabilize its structure, helps in bacterial resistance in an acidic environment such as the stomach, and helps in bacterial colonization in the host, protecting them from host immune responses [157]. Urease activity is crucial for *H. pylori* colonization in the stomach as it neutralizes gastric acids and allows the bacteria to survive in hostile environments [158], as shown in Table 3. Ni is also immersed in regulating genes involved in energy metabolism and DNA repair in *H. pylori* [159]. 

Recent research studies have shown Co’s decisive role in the physiology and virulence of several bacterial pathogens. For example, in *P. aeruginosa,* Co is required to express virulence factors, including pyoverdine, a siderophore that facilitates the uptake of Fe by the bacterium. Pyoverdine is also integrated into biofilm formation, a key mechanism for *P. aeruginosa’s* survival and pathogenesis [160,161]. Co is also incorporated in regulating genes involved in energy metabolism and resistance to oxidative stress in *P*. *aeruginosa* [162]. In addition, Co is essential for the activity of Co-dependent enzymes, such as nitrile hydratase, involved in the metabolism of nitriles and cobalamin (vitamin B12)-dependent enzymes critical for various cellular processes, including DNA synthesis and methionine metabolism [163]. Co also plays an imperative role in antibiotic resistance mechanisms in several pathogenic bacteria such as *S. aureus*, *Escherichia coli*, and *K. pneumoniae*. Metalloenzymes such as β-lactamases and aminoglycoside-modifying enzymes need Co for their proper function. These enzymes can degrade the toxic nature of antimicrobial agents, which inhibit their growth [164]. The importance of metals in bacterial pathogens’ virulence and antibiotic resistance underscores the potential of metal-related therapies to combat bacterial infections.

## 4. Interactions between Metals and Insects/Microbial Pathogens

Trace metals as micronutrients are required by all living organisms for their survival and physicochemical processes. As discussed above, microorganisms, plants, and animals compete to obtain these micronutrients to fulfill their needs. Likewise, insects and microbial pathogens also interact with other to acquire trace metals in the required concentrations. Therefore, these metals play an essential role in the association between living things. The need for these micronutrients and their physiological functions in microorganisms have been discussed in Section 3. A recent study cites that insects have evolved several mechanisms to acquire, transport, and regulate metal levels to combat microbial infections [174]. For instance, Fe is critical for the immune response of insects against microbial infections. Insects can sequester Fe from hemolymph (insect blood) and store it in tissues to limit the availability of Fe to invading pathogens [175]. Insects can also produce antibacterial peptides that require Fe for their activity, underlying the importance of Fe for the immune response [80]. In addition, Fe can also affect the composition of insect-associated microbial communities. They also have been shown to harbor various microorganisms, including bacteria, fungi, and viruses, which can have both beneficial and harmful effects on their hosts [176]. However, the unavailability of Fe can affect the growth and survival of these microorganisms and potentially alter their interactions with the insect host.

Another trace metal, such as Cu, is also essential for the immune response of insects against microbial infections [177]. Insects can produce Cu-binding proteins and transporters to remove Cu from the environment and limit its availability to invading pathogens. Cu regulates several immune genes in insects, including those encoding antimicrobial peptides, homeostasis, and proteins in response to oxidative stress that can occur during the immune response and other physiological processes [178]. In addition, Cu has been shown to upregulate the expression of genes encoding antimicrobial peptides (AMPs), which are critical effectors of the insect’s innate immune system. AMPs are small cationic peptides that can kill various microbial pathogens by disrupting their cell membranes [179].

Interestingly, one of the well-known trace minerals, Zn, is also required for the growth, development, and immune function of insects and microbial pathogens. Zn may have positive and negative effects on these organisms, depending on the concentration and method of application. Insects can produce Zn-binding proteins and transporters to regulate Zn levels and limit their availability to invading pathogens [180]. Glavinic et al. showed that supplementing honeybees with Zn can increase their resistance to specific pathogens such as *Nosema ceranae* [181]. However, high Zn concentrations can also be toxic to insects, diversely affecting their growth and survival. Zn can also positively affect the growth and virulence of certain bacteria, such as *P. aeruginosa* and *S. pneumonia* [182]. However, it can inhibit the growth of certain bacteria, such as *Salmonella typhimurium* and *E. coli*. Microbial pathogens have also evolved mechanisms to acquire and regulate metal levels to induce insect infection. For example, some bacterial pathogens can produce siderophores to remove Fe from the insect host. In contrast, some fungal pathogens can produce Zn-binding proteins and transporters to regulate Zn levels for their growth and survival [183]. Overall, the interactions between metals and insects/microbial pathogens are complex and diverse, and further research is needed to fully understand the underlying mechanisms and implications for host–pathogen interactions.

## 5. Mechanisms of Competition between Insects and Microbial Pathogens for Essential Metals

The competition for metals such as Fe, Cu, and Zn is critical to interacting with insects and microbial pathogens [174,184]. Both insects and pathogens require these metals for their growth and survival, and they have evolved multiple mechanisms to acquire and regulate metal levels. One of the primary mechanisms of competition for metals is the production of metal-binding proteins and transporters [185]. Insects can produce metal-binding proteins and transporters to regulate metal levels and limit their availability to invading pathogens. For example, insects can produce metallothionein proteins that bind to metals such as Zn and Cd, restricting their availability to invading pathogens [186].

Similarly, insects can produce ferritin proteins that sequester Fe, limiting their availability to invading pathogens. Microbial pathogens can also produce metal-binding proteins and transporters to remove metals from the insect host, as some bacterial pathogens can produce siderophores that bind to Fe and transport it into the bacterial cell [187]. Analogously, some fungal pathogens can produce Zn-binding proteins and transporters to grab Zn from the insect host [188].

Insects and microbial pathogens both compete for metals by regulating genes for metal uptake and metabolism. For example, insects may upregulate metal acquisition and metabolism genes in response to metal limitation, while microbial pathogens may also upregulate metal uptake and metabolism genes in response to metal availability [86,189]. Collectively, these micronutrients and elements have complex interactions with insects and microbial pathogens. While they are integral for proper insect development and immune function, they may also have antimicrobial properties that can help protect insects from pathogens.

## 6. Metals That Limit Microbial Growth and Virulence

Metals can limit microbial growth and virulence by affecting several biological processes, such as the reaction to oxidative stress, energy metabolism, and DNA synthesis [190]. Here are some exemplifications: Fe confinement can limit the growth and virulence of many bacterial pathogens. For example, in the bacterial pathogen *P. aeruginosa*, Fe limitation can reduce the expression of virulence factors such as siderophores and exotoxins and limit the growth of the bacterium. Fe limitation can also activate the expression of genes involved in the oxidative stress response, which can further restrict the growth and purulence of the bacterium [191,192]. The confinement of Cu can exert growth-limiting and virulence-reducing effects on various microbial pathogens. For example, in the fungal pathogen *C. albicans*, the unavailability of Cu can reduce the expression of virulence factors such as adhesins and invasins and limit fungal growth [150]. The limitation of Cu can induce the activation of gene expression related to the oxidative stress response, thereby restricting fungal development and diminishing virulence [193].

The proliferation and pathogenicity of diverse microbial pathogens can be compromised without an optimum concentration of Zn. In the case of the bacterial pathogen *E. coli*, Zn limitation can reduce the expression of virulence factors such as adhesins and fimbriae and activate the expression of genes involved in response to oxidative stress, which limits the sprout of the bacterium [194,195]. The absence of Mn has been demonstrated to significantly affect microbial physiological properties, emphasizing the importance of Mn in microbial systems. Mn in a low concentration can lead to the downregulation of genes encoding virulence factors, while increased Mn availability can lead to their upregulation [196]. In addition, another trace metal, such as Mo, can affect bacterial physiology in several ways. For example, the concentration of Mo beyond the optimum requirement can lead to changes in bacterial metabolism, including a shift toward anaerobic respiration and altered expression of genes involved in energy metabolism [197]. There is ample room for further progress in determining how metals limit microbial growth and virulence, which is necessary for developing new strategies to combat microbial infections. Targeting metal acquisition and metabolism pathways in microbial pathogens may be a promising approach for developing novel antimicrobial therapies. Metal supplementation can also be a beneficial adjunctive therapy to enhance the host’s immune response against microbial infections [86,198].

## 7. Microorganisms Require Trace Metals for Their Pathogenicity Enhancements

Various pathogenic microorganisms need different trace elements for their physiological functions to improve their pathogenicity mechanisms; for example, Fe is required by some microbes that help in enhancing the expression of virulent genes. In *Yersinia pestis*, Fe boosts the face of type III secretory system (T3SS) and type IV secretory system (T4SS). Similarly, Fe promotes these systems in *Legionella pneumophila*, enabling the bacterium to transport toxins into the host cells [199]. T3SS is a complex molecular machinery that spans the bacterial cell envelope, allowing the bacterium to transport effector proteins into host cells. These effector proteins can trigger host cell signaling pathways, disrupting cellular processes and ultimately promoting the survival and growth of the pathogen in the host [200]. In addition, Fe can stimulate the expression of sundry other virulence factors in different microbial pathogens. Likewise, Fe can promote the production of adhesins and toxins in pathogens such as *S. aureus* and *E. coli*. This association between Fe and the pathogens allows them to enhance their virulence and better exploit host systems [201].

In contrast, Cu is considered to be a natural antimicrobial agent that has been deployed to control microbial growth for centuries. Microbial pathogens have evolved multiple resistant mechanisms to counteract the toxic effects of Cu and utilize it for their survival and vigilance [202]. Some microbial pathogens leverage the regulation of Cu transport and efflux systems to negotiate with Cu. Microorganisms can also rely on Cu as a core nutrient, prompting the development of specific transport mechanisms to absorb Cu from their ambiances. Additionally, particular pathogens possess efflux pumps capable of expelling excess Cu, thereby fostering against potential toxicity [203]. In addition, some pathogens produce antioxidant enzymes, such as superoxide dismutase and catalase, that can neutralize the ROS generated by Cu. ROS can damage cellular components, including DNA, proteins, and lipids, and lead to cell death. Therefore, these pathogens can counteract ROS activity by utilizing these enzymes to neutralize ROS and protect the cell [204]. It can also enhance the response to oxidative stress in several microbial pathogens, including *P. aeruginosa* and *Salmonella enterica* [205].

Moreover, Zn can liaise with microbial pathogens by promoting the expression of virulent genes and enhancing their ability to evade host immune cells, e.g., the bacterial pathogen *S. pneumoniae* fosters the face of the pneumolysin toxin in the presence of Zn, which is the main toxin in establishing pneumococcal infections [154,206]. Alternatively, Zn has been reported to promote the expression of other pathogenic determinants, such as adhesion molecules and invasive factors, in various microbial pathogens, including *C. albicans* [207] and *S. aureus* [208]. It can also evade the host’s immune response to favor microbial infections. Mo can act as a cofactor for various enzymes, including nitrogenases, nitrate reductases, and sulfite oxidases involved in nitrogen fixation, nitrogen assimilation, and sulfur metabolism [209]. The pathogenic bacterium *P. aeruginosa* is a highly pathogenic and persistent microbe that utilizes Mo for its physiological functions. Mo is essential for expressing multiple pathogenic elements, including the siderophore, ferricrocin, pyoverdine, the enzyme catalase–peroxidase, and protease LasB, as evidenced by numerous investigations [210]. Another study examined the role of Mo in the pathogenicity of the fungus *Aspergillus fumigatus* [211]. These mechanisms through which trace metals and microbial pathogens work together are complex and diverse, and understanding these interactions is critical for developing effective strategies to control and prevent microbial infections.

## 8. Pathogens Use Metals to Evade the Host’s Immune Response

Pathogens exhibit creative strategies to escape the host immune response, often employing metals to change their physiology. Most bacterial pathogens depend on Fe for their growth and survival [212]. These cunning invaders utilize intricate Fe capture mechanisms to extract it from the host, thereby restricting its availability to immune cells, thus undermining the host’s defenses [39,213]. Several pathogens can also produce Fe-binding proteins, such as transferrin-binding and lactoferrin-binding proteins, to sequester Fe from the host [214]. It can limit the availability of Fe to host immune cells and impair their function. As reported by various scientific analyses, Cu is involved in the oxidative stress response of host immune cells, such as neutrophils and macrophages. Pathogens can employ Cu efflux pumps to diminish Cu levels within host immune cells, impairing their oxidative capacity [86,215]. Some pathogens can also produce Cu-binding proteins such as CopA and CopB in *P. aeruginosa* to sequester Cu from the host and restrict its availability to host immune cells [216,217].

Multivalent metals like Zn are entangled in multiple immune responses, including the regulation of cytokine production and the activation of immune cells. Pathogens can utilize Zn efflux pumps to decrease Zn levels in host immune cells and impair their function [218]. Specific pathogens, like *E. coli*, can generate Zn-binding proteins (e.g., ZnuA and ZinT). These proteins help the pathogens to seize and limit the availability of Zn from the host, consequently impeding the function of immune cells [219]. Specific bacterial pathogens, like *H. pylori,* responsible for gastric ulcers and cancer, employ Ni to evade their host’s immune response. Researchers found that the pathogen can take Ni from the host environment and use it to produce enzymes called ureases that can break down urea and release ammonia [220]. This ammonia can neutralize the stomach’s acidic environment and create a more favorable environment for the colonization of the pathogen. In addition, ureases produced by *H. pylori* can interact with the host’s immune cells and impair their function, allowing the pathogen to escape the host’s immune responses [221]. In addition, another bacterial pathogen, *S. aureus,* also utilizes Ni to resist the host immune response. *S. aureus* can take Ni from the host environment and use it to produce a surface protein called IsdA, which reduces microbial cellular hydrophobicity and decreases bacterial cellular hydrophobicity, posing them to resist the antibacterial human skin fatty acids and peptides [222]. Investigating the utilization of these miscellaneous metals via pathogens sheds light on how they evade the host’s immune response. This knowledge is crucial for devising novel approaches to combat microbial infections. In summary, this review underscores the significance of metals as vital facilitators in insect–microbe interactions, paving the way for exciting prospects in future research endeavors within this domain.

## 9. Implications and Applications

### 9.1. Development of Novel Insecticides and Antimicrobial Therapies

In scientific innovation, metals are vital in developing new insecticides and antimicrobial treatments. Researchers utilize metals like Cu and Zn to create robust solutions against pests and diseases [223]. Cu disrupts fungal invaders, protecting precious crops from their insatiable appetite [224]. Furthermore, Zn exposes its elemental ability, engaging in a microbial duel, vanquishing the bacteria, fungi, and viruses that threaten our delicate balance [225]. Novel insecticides and antimicrobial therapies are crucial for agriculture and public health. However, resistance to traditional chemicals is a growing concern [226]. Developing novel insecticides and antimicrobial treatments targeting specific biological pathways or mechanisms can help address these challenges. However, the safety and efficacy of these novel compounds must be thoroughly evaluated to ensure they do not pose unintended risks to the environment or human health [227]. However, this development will improve the innovation of novel insecticides and antimicrobial therapies.

### 9.2. Promoting Agricultural Sustainability

**Figure 5:**
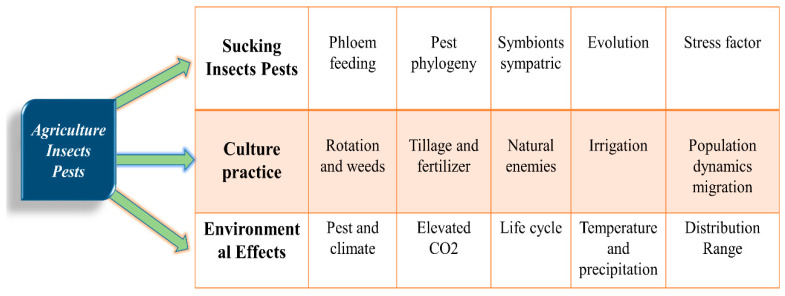
Schematic representation of insect pest control strategies using the hypothetical model of standard land preparation such as rotation, fallow, soil treatment, and irrigation with insect pest control strategies.

Several insect pests and microbial pathogens are major crop threats, resulting in significant economic losses [228]. Understanding the role of metals in the interaction between insect and microbial pathogens can lead to the development of sustainable and environmentally friendly pest and disease control strategies that target metal uptake and metabolic pathways in these organisms [229]. However, excessive use of metals in agricultural systems can negatively impact insect and microbial populations. Contradicting reports about high concentrations of Cu and Zn can lead to the selection of metal-resistant bacteria and disruption of microbial communities that play essential roles in nutrient cycling and soil health. Insects can also develop resistance to metals, reducing the effectiveness of metal-based pesticides and making plants vulnerable to insect damage [230]. Therefore, promoting agricultural sustainability requires balancing the benefits and risks of using metals in agriculture. It can be achieved by developing alternative pest management strategies, such as biological control and integrated pest management, that minimize the use of metals and promote the natural regulation of insect and microbial populations [231]. Figure 5 illustrates the intricate interactions between soil, plant, and microbes and how fumigants or drought stress can kill beneficial bacteria in the soil. Furthermore, exploring interactions between metals, insects, and microbial pathogens can provide insights into the mechanisms of insect immunity and microbial pathogenesis, leading to the development of novel and sustainable crop protection strategies.

### 9.3. Potential Applications for Pest and Pathogen Control

The role of metals in insect and microbial pathogen interactions has received significant attention due to their potential applications in pest and pathogen control. Metal ions, such as Cu, Zn, and Fe, play an essential role in insect physiology, including growth, development, and reproduction [232]. In addition, some metals exhibit antimicrobial properties that can help control insect microbial pathogens. One pest management method is biological control, which involves using natural enemies to control pests. Several biological control agents, such as entomopathogenic fungi and bacteria, have been effective against pests and pathogens [233]. Metals play a crucial role in these biological control agents’ survival, virulence, and interplay with pests and pathogens.

Metal homeostasis and redox signaling mechanisms are pivotal in the responses of insects and microbial pathogens to environmental stressors, including exposure to toxic metals [232]. Many models about nanoparticles, biomimicry, and bioremediation are emerging research areas that hold promise for developing new approaches to pest and pathogen control [234]. These approaches rely on metals’ unique properties and interactions with biological systems to develop effective control measures. 

## 10. Critical Observation and Further Research

Metals play a crucial role in microbial–insect interactions, but their precise mechanisms and specific functions are largely unknown. Further research is needed to better understand the interactions between metals, insects, and microbial pathogens to develop strategies to combat insect-borne diseases. In addition, research is required to explore the full potential of metals in pest and disease control and their effectiveness and safety in these applications. Insects are an essential food source for humans and animals but are responsible for spreading various diseases. Interactions between insects and microbial pathogens can be complex, involving multiple species and multiple routes of transmission. Therefore, research is also needed to better understand the mechanisms of information and the potential impact of these interactions on human and animal health. Insects are often the primary hosts of microbial pathogens, making them more susceptible to the transmission of pathogens. Effective control of insect-borne diseases requires an integrated approach that includes insect control, vector surveillance, and strategies to reduce human–animal contact. Climate change will likely significantly impact insect populations and the spread of microbial diseases. Therefore, future research is needed to understand the impact of climate change and other environmental factors on the interactions between insects and microbial pathogens.

## 11. Conclusions

In summary, metals are crucial in insects and microbial pathogens. In insects, metals such as Cu, Fe, Zn, Mn, Ni, Co, and Mo are essential for vital biological processes such as respiration, immune response, and enzymatic reactions. In microbial pathogens, metals such as Fe and Mn are critical in enhancing their virulence factors, as they are involved in oxidative stress defense, metabolism, and gene regulation. However, the availability and regulation of metals in insects and microbial pathogens can significantly affect their health and survival. Therefore, exploring the role of metals in these organisms can provide valuable insights into their biology and potential targets for developing new treatments and control strategies. Research into the role of metals in insect and microbial pathogens can have significant implications for developing new approaches to combat these organisms.

However, since metals are critical to the survival and virulence of microbial pathogens, targeting their metal acquisition and regulatory mechanisms could potentially lead to the development of novel antimicrobial agents. In addition, understanding how insects take up and regulate metals could lead to the development of new insecticides that interfere with their vital biological processes. The reason for studying the interactions between metals, insects, and microbial pathogens can shed light on how these organisms respond to environmental stressors such as changes in metal availability. This knowledge can be used to develop new strategies to control insect pests and microbial pathogens under different environmental conditions. Overall, understanding the role of metals in insect and microbial pathogens can lead to developing new, more effective strategies to control these organisms, which could have significant implications for agriculture, public health, and environmental management.

## Figures and Tables

**Table 1:** Interaction of plants with insects and microbes through Fe.

Fe	Role in Plants	Role in Insects	Role in Microbes
Absorption	Plants take up Fe through their roots and transport it to different plant parts for use in metabolic processes [51].	Insects acquire Fe from their diet, including plant material [52].	Microbes acquire Fe from their environment for growth and metabolism [53].
Defense	Fe produces defensive compounds in plants, such as phytochelatin and lignin [54].	Insects can use Fe-binding proteins to sequester Fe from their diet and prevent it from being used by pathogens [55].	Some microbes can produce Fe-chelating compounds called siderophores, competing with plants and insects for Fe [56].
Signaling	Fe is involved in signaling pathways in plants, e.g., the regulation of gene expression and responses to stress [57].	Fe can act as a signaling molecule in insects, affecting behavior and development [58].	Fe availability can also affect microbial gene expression and behavior [59].
Regulation	Plants regulate Fe acquisition and transport through a complex system of genes and proteins [60].	Insects require a certain amount of Fe for proper growth and development, but excess Fe can be toxic [61].	Microbes have evolved mechanisms to sense and respond to Fe availability in their environment [62].

**Table 2:** Condensing the role of metals in the defensive mechanisms of insects.

Insects	Metals	Defensive Mechanisms	References
Ironclad beetle	Fe	Hardens exoskeleton for protection against predators.	[120]
Bombardier Beetle	Cu	Copper enzymes facilitate the expulsion of toxic sprays.	[121]
Leafhopper	Zn	Protection of exoskeleton for defense against predators.	[122]
Silverfish	Ag	Antimicrobial defense, silver scales provide camouflage and protection.	[123]
Sweat Bees	Hg	It uses mercury to deter parasites and pathogens.	[124]
Fireflies	Co	Cobalt ions play a role in the production of bioluminescence.	[125]
Nickel-Eating Moth	Ni	Nickel detoxification enables feeding on nickel-rich plants.	[126]
Selenium-Superior Fly	Se	Selenium detoxification offers resistance to toxic plants.	[127]

**Table 3:** Summarizing specific microbes, virulent factors, and metals associated with their roles.

Microbes	Virulence Factor	Role of Trace Metals	References
*Candida albicans*	Phospholipase enzymes	Ca: Activates and stabilizes phospholipase enzymes, also secretes candida lysin.	[165]
*Pseudomonas aeruginosa*	Pyocyanin	Fe: Crucial for pyocyanin synthesis, a virulent pigment.	[166,167]
*Streptococcus pneumoniae*	Pneumolysin	Zn: Activates pneumolysin, causing host cell damage.	[168]
*Helicobacter pylori*	Urease	Ni: Essential for urease activity, aiding survival in an acidic stomach.	[169]
*Escherichia coli*	Shiga toxin	Fe, Zn, Cu, and Mg: Essential for toxin production and stability.	[170,171]
*Staphylococcus aureus*	Coagulase	Ca: Activates coagulase and aids in blood clotting.	[172]
*Vibrio cholerae*	Cholera toxin	Zn: Stabilizes cholera toxin structure for practical function.	[173]

## References

[1] Zhang L., Su Q.F., Wang L.S., Lv M.W., Hou Y.X., Li S.S. (2022). Linalool: A ubiquitous floral volatile mediating the communication between plants and insects. J. Syst. Evol..

[2] Leitão J.H. (2020). Microbial Virulence Factors. IJMS.

[3] Biere A., Bennett A.E. (2013). Three-way interactions between plants, microbes and insects. Functional.

[4] Zhang Q., Chen X., Xu C., Zhao H., Zhang X., Zeng G., Qian Y., Liu R., Guo N., Mi W. (2019). Horizontal gene transfer allowed the emergence of broad host range entomopathogens. Proc. Natl. Acad. Sci. USA.

[5] Guo Z., Guo L., Bai Y., Kang S., Sun D., Qin J., Ye F., Wang S., Wu Q., Xie W. (2023). Retrotransposon-mediated evolutionary rewiring of a pathogen response orchestrates a resistance phenotype in an insect host. Proc. Natl. Acad. Sci. USA.

[6] Anand U., Pal T., Yadav N., Singh V.K., Tripathi V., Choudhary K.K., Shukla A.K., Sunita K., Kumar A., Bontempi E. (2023). Current Scenario and Future Prospects of Endophytic Microbes: Promising Candidates for Abiotic and Biotic Stress Management for Agricultural and Environmental Sustainability. Microb. Ecol..

[7] Perveen N., Muhammad K., Muzaffar S.B., Zaheer T., Munawar N., Gajic B., Sparagano O.A., Kishore U., Willingham A.L. (2023). Host-pathogen interaction in arthropod vectors: Lessons from viral infections. Front. Immunol..

[8] Gebre S.H. (2023). Bio-inspired Synthesis of Metal and Metal Oxide Nanoparticles: The Key Role of Phytochemicals. J. Clust. Sci..

[9] Page M.G.P. (2019). The Role of Iron and Siderophores in Infection, and the Development of Siderophore Antibiotics. Clin. Infect. Dis..

[10] Valko M., Rhodes C.J., Moncol J., Izakovic M., Mazur M. (2006). Free radicals, metals and antioxidants in oxidative stress-induced cancer. Chem. Biol. Interact..

[11] Zhang Y.-Y., Li X.-S., Ren K.-D., Peng J., Luo X.-J. (2023). Restoration of metal homeostasis: A potential strategy against neurodegenerative diseases. Ageing Res. Rev..

[12] Dharmaraja A.T. (2017). Role of Reactive Oxygen Species (ROS) in Therapeutics and Drug Resistance in Cancer and Bacteria. J. Med. Chem..

[13] Schrand A.M., Rahman M.F., Hussain S.M., Schlager J.J., Smith D.A., Syed A.F. (2010). Metal-based nanoparticles and their toxicity assessment. Wiley Interdiscip. Rev. Nanomed. Nanobiotechnol..

[14] Lin Y.F., Aarts M.G. (2012). The molecular mechanism of zinc and cadmium stress response in plants. Cell. Mol. Life Sci. CMLS.

[15] Thallaj N. (2023). Review of a Few Selected Examples of Intermolecular Dioxygenases Involving Molecular Oxygen and Non-Heme Iron Proteins. Int. J. Adv. Parmacutical Sci. Res. (IJAPSR).

[16] Rout G.R., Sahoo S. (2015). ROLE OF Iron in Plant Growth and Metabolism. Rev. Agric. Sci..

[17] Stijlemans B., Beschin A., Magez S., Van Ginderachter J.A., De Baetselier P. (2015). Iron homeostasis and Trypanosoma brucei associated immunopathogenicity development: A battle/quest for iron. BioMed Res. Int..

[18] Aziz D.A.A., Penyelidikan P.P. (2001). The Development and Optimization of Processes for The expression of Sialylated Recombinant Human Therapeutic Glycoprotein in Insect Cell-Baculovirus System.

[19] Macaluso G., Grippi F., Di Bella S., Blanda V., Gucciardi F., Torina A., Guercio A., Cannella V. (2023). A Review on the Immunological Response against Trypanosoma cruzi. Pathogens.

[20] Ganz T. (2011). Hepcidin and iron regulation, 10 years later. Blood J. Am. Soc. Hematol..

[21] Ganz T., Nemeth E. (2012). Hepcidin and iron homeostasis. Biochim. Et Biophys. Acta (BBA)-Mol. Cell Res..

[22] Drakesmith H., Nemeth E., Ganz T. (2015). Ironing out ferroportin. Cell Metab..

[23] Winter W.E., Bazydlo L.A., Harris N.S. (2014). The molecular biology of human iron metabolism. Lab. Med..

[24] Kalinowski D.S., Richardson D.R. (2010). Cellular and molecular biology of iron-binding proteins. Cellular and Molecular Biology of Metals.

[25] Tandara L., Salamunić I. (2012). Iron metabolism: Current facts and future directions. Biochem. Medica.

[26] Smith A., McCulloh R.J. (2015). Hemopexin and haptoglobin: Allies against heme toxicity from hemoglobin not contenders. Front. Physiol..

[27] Payne S.M. (1993). Iron acquisition in microbial pathogenesis. Trends Microbiol..

[28] Chu B.C., Garcia-Herrero A., Johanson T.H., Krewulak K.D., Lau C.K., Peacock R.S., Slavinskaya Z., Vogel H.J. (2010). Siderophore uptake in bacteria and the battle for iron with the host; a bird’s eye view. Biometals.

[29] Marchetti M., De Bei O., Bettati S., Campanini B., Kovachka S., Gianquinto E., Spyrakis F., Ronda L. (2020). Iron metabolism at the interface between host and pathogen: From nutritional immunity to antibacterial development. Int. J. Mol. Sci..

[30] Barton L.L. (2005). Transmembrane Movement: Mechanisms and Examples. Struct. Funct. Relatsh. Prokaryotes.

[31] Wilson B.R., Bogdan A.R., Miyazawa M., Hashimoto K., Tsuji Y. (2016). Siderophores in iron metabolism: From mechanism to therapy potential. Trends Mol. Med..

[32] Wang W., Lu Y., Wang Y., Zhang Y., Xia B., Cao J. (2020). Siderophores induce mitophagy-dependent apoptosis in platelets. Ann. Transl. Med..

[33] Dolezal T., Krejcova G., Bajgar A., Nedbalova P., Strasser P. (2019). Molecular regulations of metabolism during immune response in insects. Insect Biochem. Mol. Biol..

[34] Scott Z.W., Choi S.-r., Britigan B.E., Narayanasamy P. (2023). Development of Gallium (III) as an Antimicrobial Drug Targeting Pathophysiologic Iron Metabolism of Human Pathogens. ACS Infect. Dis..

[35] Laranjeira-Silva M.F., Hamza I., Pérez-Victoria J.M. (2020). Iron and heme metabolism at the leishmania–host interface. Trends Parasitol..

[36] Dolan S.K. (2023). Illuminating Siderophore Transporter Functionality with Thiopeptide Antibiotics. mBio.

[37] Sánchez-Jiménez A., Marcos-Torres F.J., Llamas M.A. (2023). Mechanisms of iron homeostasis in Pseudomonas aeruginosa and emerging therapeutics directed to disrupt this vital process. Microb. Biotechnol..

[38] Banerjee S., Farhana A., Ehtesham N.Z., Hasnain S.E. (2011). Iron acquisition, assimilation and regulation in mycobacteria. Infect. Genet. Evol..

[39] Rementeria A., López-Molina N., Ludwig A., Vivanco A.B., Bikandi J., Pontón J., Garaizar J. (2005). Genes and molecules involved in Aspergillus fumigatus virulence. Rev. Iberoam Micol..

[40] Kita K., Nihei C., Tomitsuka E. (2003). Parasite Mitochondria as Drug Target: Diversity and Dynamic Changes During the Life Cycle. Curr. Med. Chem..

[41] Huber D., Römheld V., Weinmann M. (2012). Relationship between nutrition, plant diseases and pests. Marschner’s Mineral Nutrition of Higher Plants.

[42] Haschka D., Hoffmann A., Weiss G. (2021). Iron in immune cell function and host defense. Seminars in Cell & Developmental Biology, 2021.

[43] Khan S., Subhan F., Haleem K.S., Khattak M.N.K., Khan I., Sultan T., Tauseef I. (2018). Impact of plant growth-promoting rhizobacteria on yield and disease control of Nicotiana tabacum. Arch. Biol. Sci..

[44] Iwama R.E., Moran Y. (2023). Origins and diversification of animal innate immune responses against viral infections. Nat. Ecol. Evol..

[45] Pérez-Montaño F., Alias-Villegas C., Bellogín R.A., del Cerro P., Espuny M.R., Jiménez-Guerrero I., López-Baena F.J., Ollero F., Cubo T. (2014). Plant growth promotion in cereal and leguminous agricultural important plants: From microorganism capacities to crop production. Microbiol. Res..

[46] Vidhyasekaran P. (2015). Plant Hormone Signaling Systems in Plant Innate Immunity.

[47] García M.J., Lucena C., Romera F.J. (2021). Ethylene and Nitric Oxide Involvement in the Regulation of Fe and P Deficiency Responses in Dicotyledonous Plants. Int. J. Mol. Sci..

[48] Maurer F., Müller S., Bauer P. (2011). Suppression of Fe deficiency gene expression by jasmonate. Plant Physiol. Biochem..

[49] Brumbarova T., Bauer P., Ivanov R. (2015). Molecular mechanisms governing Arabidopsis iron uptake. Trends Plant Sci..

[50] Cui Y., Chen C.-L., Cui M., Zhou W.-J., Wu H.-L., Ling H.-Q. (2018). Four IVa bHLH Transcription Factors Are Novel Interactors of FIT and Mediate JA Inhibition of Iron Uptake in Arabidopsis. Mol. Plant.

[51] Hell R., Stephan U.W. (2003). Iron uptake, trafficking and homeostasis in plants. Planta.

[52] Hu L., Mateo P., Ye M., Zhang X., Berset J.D., Handrick V., Radisch D., Grabe V., Köllner T.G., Gershenzon J. (2018). Plant iron acquisition strategy exploited by an insect herbivore. Science.

[53] Ratledge C., Dover L.G. (2000). Iron Metabolism in Pathogenic Bacteria. Annu. Rev. Microbiol..

[54] Yadav B., Dubey R., Gnanasekaran P., Narayan O.P. (2021). OMICS approaches towards understanding plant’s responses to counterattack heavy metal stress: An insight into molecular mechanisms of plant defense. Plant Gene.

[55] Rodríguez-García C., Heerman M.C., Cook S.C., Evans J.D., DeGrandi-Hoffman G., Banmeke O., Zhang Y., Huang S., Hamilton M., Chen Y.P. (2021). Transferrin-mediated iron sequestration suggests a novel therapeutic strategy for controlling Nosema disease in the honey bee, Apis mellifera. PLoS Pathog..

[56] Pahari A., Pradhan A., Nayak S.K., Mishra B.B. (2017). Bacterial Siderophore as a Plant Growth Promoter. Microb. Biotechnol..

[57] Schmidt W. (2003). Iron solutions: Acquisition strategies and signaling pathways in plants. Trends Plant Sci..

[58] Venturi V., Keel C. (2016). Signaling in the Rhizosphere. Trends Plant Sci..

[59] Green J., Rolfe M.D., Smith L.J. (2014). Transcriptional regulation of bacterial virulence gene expression by molecular oxygen and nitric oxide. Virulence.

[60] Rodrigues W.F.C., Lisboa A.B.P., Lima J.E., Ricachenevsky F.K., Del-Bem L.E. (2023). Ferrous iron uptake via IRT1/ZIP evolved at least twice in green plants. New Phytol..

[61] Gorman M.J. (2023). Iron Homeostasis in Insects. Annu. Rev. Entomol..

[62] Andrews S.C., Robinson A.K., Rodríguez-Quiñones F. (2003). Bacterial iron homeostasis. FEMS Microbiol. Rev..

[63] Nawaz A., Rehman H.U., Usman M., Wakeel A., Shahid M.S., Alam S., Sanaullah M., Atiq M., Farooq M. (2023). Nanobiotechnology in crop stress management: An overview of novel applications. Discov. Nano.

[64] Mwangi M.N., Oonincx D.G., Stouten T., Veenenbos M., Melse-Boonstra A., Dicke M., Van Loon J.J. (2018). Insects as sources of iron and zinc in human nutrition. Nutr. Res. Rev..

[65] Jones M.W.M., de Jonge M.D., James S.A., Burke R. (2015). Elemental mapping of the entire intact Drosophila gastrointestinal tract. JBIC J. Biol. Inorg. Chem..

[66] Kambe T., Tsuji T., Hashimoto A., Itsumura N. (2015). The Physiological, Biochemical, and Molecular Roles of Zinc Transporters in Zinc Homeostasis and Metabolism. Physiol. Rev..

[67] Navarro J.A., Schneuwly S. (2017). Copper and Zinc Homeostasis: Lessons from Drosophila melanogaster. Front. Genet..

[68] Groth C., Sasamura T., Khanna M.R., Whitley M., Fortini M.E. (2013). Protein trafficking abnormalities in Drosophila tissues with impaired activity of the ZIP7 zinc transporter Catsup. Development.

[69] Guo Z., Qin J., Zhou X., Zhang Y. (2018). Insect Transcription Factors: A Landscape of Their Structures and Biological Functions in Drosophila and beyond. Int. J. Mol. Sci..

[70] González-Santoyo I., Córdoba-Aguilar A. (2011). Phenoloxidase: A key component of the insect immune system. Entomol. Exp. Appl..

[71] Kanost M., Gorman M. (2008). Phenoloxidases in insect immunity. Insect Immunol..

[72] Li Y., Wang Y., Jiang H., Deng J. (2009). Crystal structure of *Manduca sexta* prophenoloxidase provides insights into the mechanism of type 3 copper enzymes. Proc. Natl. Acad. Sci. USA.

[73] Xiao G. (2022). Molecular physiology of zinc in Drosophila melanogaster. Curr. Opin. Insect Sci..

[74] Balamurugan K., Egli D., Hua H., Rajaram R., Seisenbacher G., Georgiev O., Schaffner W. (2007). Copper homeostasis in Drosophila by complex interplay of import, storage and behavioral avoidance. EMBO J..

[75] Selvaraj A., Balamurugan K., Yepiskoposyan H., Zhou H., Egli D., Georgiev O., Thiele D.J., Schaffner W. (2005). Metal-responsive transcription factor (MTF-1) handles both extremes, copper load and copper starvation, by activating different genes. Genes Dev..

[76] Slobodian M.R., Petahtegoose J.D., Wallis A.L., Levesque D.C., Merritt T.J. (2021). The effects of essential and non-essential metal toxicity in the Drosophila melanogaster insect model: A review. Toxics.

[77] Bahadorani S., Mukai S., Egli D., Hilliker A.J. (2010). Overexpression of metal-responsive transcription factor (MTF-1) in Drosophila melanogaster ameliorates life-span reductions associated with oxidative stress and metal toxicity. Neurobiol. Aging.

[78] Wintergerst E.S., Maggini S., Hornig D.H. (2006). Immune-enhancing role of vitamin C and zinc and effect on clinical conditions. Ann. Nutr. Metab..

[79] Stączek S., Cytryńska M., Zdybicka-Barabas A. (2023). Unraveling the Role of Antimicrobial Peptides in Insects. Int. J. Mol. Sci..

[80] Hrdina A., Iatsenko I. (2022). The roles of metals in insect–microbe interactions and immunity. Curr. Opin. Insect Sci..

[81] McHenry C.S. (2011). DNA replicases from a bacterial perspective. Annu. Rev. Biochem..

[82] Ishida T. (2018). Antiviral activities of Zn^2+^ ions for viral prevention, replication, capsid protein in intracellular proliferation of viruses. World Sci. News.

[83] Kuznetsova A.A., Fedorova O.S., Kuznetsov N.A. (2022). Structural and molecular kinetic features of activities of DNA polymerases. Int. J. Mol. Sci..

[84] Lee J.Y., Chang C., Song H.K., Moon J., Yang J.K., Kim H.-K., Kwon S.-T., Suh S.W. (2000). Crystal structure of NAD+-dependent DNA ligase: Modular architecture and functional implications. EMBO J..

[85] Begg S.L. (2019). The role of metal ions in the virulence and viability of bacterial pathogens. Biochem. Soc. Trans..

[86] Sharma K.K., Singh D., Mohite S.V., Williamson P.R., Kennedy J.F. (2023). Metal manipulators and regulators in human pathogens: A comprehensive review on microbial redox copper metalloenzymes “multicopper oxidases and superoxide dismutases”. Int. J. Biol. Macromol..

[87] Andrés C.M.C., de la Lastra J.M.P., Juan C.A., Plou F.J., Pérez-Lebeña E. (2023). Superoxide Anion Chemistry—Its Role at the Core of the Innate Immunity. Int. J. Mol. Sci..

[88] Zaminpira S., Niknamian S. (2017). How butterfly effect or deterministic chaos theory in theoretical physics explains the main cause of cancer. EC Cancer.

[89] Stengel A. (2009). Characterisation of components and mechanisms involved in redox-regulation of protein import into chloroplasts. LMU.

[90] Isernia C., Bucci E., Leone M., Zaccaro L., Di Lello P., Digilio G., Esposito S., Saviano M., Di Blasio B., Pedone C. (2003). NMR structure of the single QALGGH zinc finger domain from the Arabidopsis thaliana SUPERMAN protein. Chembiochem.

[91] Zhu H., Chen C., Zeng J., Yun Z., Liu Y., Qu H., Jiang Y., Duan X., Xia R. (2020). Micro RNA 528, a hub regulator modulating ROS homeostasis via targeting of a diverse set of genes encoding copper-containing proteins in monocots. New Phytol..

[92] Smirnoff N., Arnaud D. (2019). Hydrogen peroxide metabolism and functions in plants. New Phytol..

[93] Iqbal N., Trivellini A., Masood A., Ferrante A., Khan N.A. (2013). Current understanding on ethylene signaling in plants: The influence of nutrient availability. Plant Physiol. Biochem..

[94] Zekri M., Obreza T.A. (2003). Plant Nutrients for Citrus Trees.

[95] Gall J.E., Boyd R.S., Rajakaruna N. (2015). Transfer of heavy metals through terrestrial food webs: A review. Environ. Monit. Assess..

[96] Gaspéri J., Ayrault S., Moreau-Guigon E., Alliot F., Labadie P., Budzinski H., Blanchard M., Muresan B., Caupos E., Cladière M. (2018). Contamination of soils by metals and organic micropollutants: Case study of the Parisian conurbation. Environ. Sci. Pollut. Res..

[97] Whitfield M. (2001). Interactions between phytoplankton and trace metals in the ocean. Advances in Marine Biology..

[98] Aparachita P. (2020). Effects of Water Soluble Zinc on Growth Performance, Immune Response, and Tissue Zinc Transporters in Nursery Pigs.

[99] Castro P.H., Lilay G.H., Assunção A.G. (2018). Regulation of micronutrient homeostasis and deficiency response in plants. Plant Micronutrient Use Efficiency.

[100] Kuliyev E. (2020). Functional and Biochemical Characterization of ZIP4 Extracellular Domain⎯Implications on Acrodermatitis Enteropathica, a Life-Threatening Genetic Disorder.

[101] Murakami M., Hirano T. (2008). Intracellular zinc homeostasis and zinc signaling. Cancer Sci..

[102] John E., Laskow T.C., Buchser W.J., Pitt B.R., Basse P.H., Butterfield L.H., Kalinski P., Lotze M.T. (2010). Zinc in innate and adaptive tumor immunity. J. Transl. Med..

[103] Rajkumar M., Bruno L.B., Banu J.R. (2017). Alleviation of environmental stress in plants: The role of beneficial *Pseudomonas* spp.. Crit. Rev. Environ. Sci. Technol..

[104] Naz T., Iqbal M.M., Fahad S., Akhtar J., Saqib M., Alamri S., Siddiqui M.H., Saud S., Khattak J.Z.K., Ali S. (2023). Bio-fortification of Two Wheat Cultivars with Iron and Zinc Through Their Soil and Foliar Application in Salt-Factored Soil: Growth, Ionic, Physiological, and Biochemical Modifications. J. Plant Growth Regul..

[105] Zehra A., Raytekar N.A., Meena M., Swapnil P. (2021). Efficiency of microbial bio-agents as elicitors in plant defense mechanism under biotic stress: A review. Curr. Res. Microb. Sci..

[106] Enebe M.C., Babalola O.O. (2019). The impact of microbes in the orchestration of plants’ resistance to biotic stress: A disease management approach. Appl. Microbiol. Biotechnol..

[107] Kahromi S., Khara J. (2021). Chitosan stimulates secondary metabolite production and nutrient uptake in medicinal plant*Dracocephalum kotschyi*. J. Sci. Food Agric..

[108] Khan T., Abbasi B.H., Khan M.A. (2018). The interplay between light, plant growth regulators and elicitors on growth and secondary metabolism in cell cultures of Fagonia indica. J. Photochem. Photobiol. B Biol..

[109] Caverzan A., Passaia G., Rosa S.B., Ribeiro C.W., Lazzarotto F., Margis-Pinheiro M. (2012). Plant responses to stresses: Role of ascorbate peroxidase in the antioxidant protection. Genet. Mol. Biol..

[110] Russo M., Petropoulos V., Molotokaite E., Cerullo G., Casazza A.P., Maiuri M., Santabarbara S. (2020). Ultrafast excited-state dynamics in land plants Photosystem I core and whole supercomplex under oxidised electron donor conditions. Photosynth. Res..

[111] Winterbourn C.C. (2020). Biological chemistry of superoxide radicals. Chemtexts.

[112] Turk H., Erdal S. (2015). Melatonin alleviates cold-induced oxidative damage in maize seedlings by up-regulating mineral elements and enhancing antioxidant activity. J. Plant Nutr. Soil Sci..

[113] Song X., Zhong Z., Gao L., Weiss B.L., Wang J. (2022). Metabolic interactions between disease-transmitting vectors and their microbiota. Trends Parasitol..

[114] Soetan K., Olaiya C., Oyewole O. (2010). The importance of mineral elements for humans, domestic animals and plants: A review. Afr. J. Food Sci..

[115] Bhatla S.C.A., Lal M., Kathpalia R., Bhatla S.C. (2018). Plant mineral nutrition. Plant Physiol. Dev. Metab..

[116] Jha C.K., Saraf M. (2015). Plant growth promoting rhizobacteria (PGPR): A review. J. Agric. Res. Dev..

[117] Rajak P., Roy S., Ganguly A., Mandi M., Dutta A., Das K., Nanda S., Sarkar S., Khatun S., Ghanty S. (2022). Protective potential of vitamin C and E against organophosphate toxicity: Current status and perspective. J. Ecophysiol. Occup. Health.

[118] Yruela I. (2009). Copper in plants: Acquisition, transport and interactions. Funct. Plant Biol..

[119] Li L., Yi H. (2012). Effect of sulfur dioxide on ROS production, gene expression and antioxidant enzyme activity in Arabidopsis plants. Plant Physiol. Biochem..

[120] Huang W., Montroni D., Wang T., Murata S., Arakaki A., Nemoto M., Kisailus D. (2022). Nanoarchitected Tough Biological Composites from Assembled Chitinous Scaffolds. Acc. Chem. Res..

[121] Zhou S., Jiang L., Dong Z. (2022). Overflow control for sustainable development by superwetting surface with biomimetic structure. Chem. Rev..

[122] Goetzke H.H., Pattrick J.G., Federle W. (2019). Froghoppers jump from smooth plant surfaces by piercing them with sharp spines. Proc. Natl. Acad. Sci. USA.

[123] Stewart R. (2002). Robin Stewart’s Chemical Free Pest Control: Hundreds of Practical & Inexpensive Ways to Control Pests without Chemicals.

[124] Gochfield M., Laumbach R. (2011). Chemical hazards. Occupational and Environemntal Health-Recognizing and Preventing Disease and Injury.

[125] Deng L. (2004). Shining the Light: Structure and Function Relationship of Calcium-Regulated Photoproteins.

[126] Waterhouse D. (1952). Studies on the digestion of wool by insects IV. Absorption and elimination of metals by Lepidopterous larvae, with special reference to the clothes moth, Tineola Bisselliella (Humm.). Aust. J. Biol. Sci..

[127] Mma A.A.F., Ahmed F., Abada M.A.M., Yassen E. (2017). Effect of spraying silicon and selenium on growth, vine nutritional status, berry setting, yield and berries quality of superior grapevines grown under sandy soil conditions i-the effect on growth and vine nutritional status. Fayoum J. Agric. Res. Dev..

[128] Bánfalvi G. (2011). Heavy metals, trace elements and their cellular effects. Cell. Eff. Heavy Met..

[129] Collin F. (2019). Chemical Basis of Reactive Oxygen Species Reactivity and Involvement in Neurodegenerative Diseases. Int. J. Mol. Sci..

[130] Zhong X., Wright J.F. (2013). Biological Insights into Therapeutic Protein Modifications throughout Trafficking and Their Biopharmaceutical Applications. Int. J. Cell Biol..

[131] Moskovitz J., Yim M.B., Chock P.B. (2002). Free radicals and disease. Arch. Biochem. Biophys..

[132] Calderon I.L., Elías A.O., Fuentes E.L., Pradenas G.A., Castro M.E., Arenas F.A., Perez J.M., Vasquez C.C. (2009). Tellurite-mediated disabling of [4Fe–4S] clusters of Escherichia coli dehydratases. Microbiology.

[133] Madkour L.H. (2019). Targeted Drug Delivery. J. Target. Drug Deliv..

[134] David S.S., O’Shea V.L., Kundu S. (2007). Base-excision repair of oxidative DNA damage. Nature.

[135] Martinez-Zamudio R., Ha H.C. (2011). Environmental epigenetics in metal exposure. Epigenetics.

[136] Zhu K.Y., Merzendorfer H., Zhang W., Zhang J., Muthukrishnan S. (2016). Biosynthesis, turnover, and functions of chitin in insects. Annu. Rev. Entomol..

[137] Tariq A., Zeng F., Graciano C., Ullah A., Sadia S., Ahmed Z., Murtaza G., Ismoilov K., Zhang Z. (2023). Regulation of Metabolites by Nutrients in Plants. Plant Ionomics: Sensing, Signaling, and Regulation.

[138] Bretscher H., O’connor M.B. (2020). The Role of Muscle in Insect Energy Homeostasis. Front. Physiol..

[139] Mattson W.J. (1980). Herbivory in relation to plant nitrogen content. Annu. Rev. Ecol. Syst..

[140] Dresen M., Valentin-Weigand P., Berhanu Weldearegay Y. (2023). Role of Metabolic Adaptation of Streptococcus suis to Host Niches in Bacterial Fitness and Virulence. Pathogens.

[141] Aggarwal S., Kumaraswami M. (2022). Managing Manganese: The Role of Manganese Homeostasis in Streptococcal Pathogenesis. Front. Cell Dev. Biol..

[142] Mitchell A., Mitchell T. (2010). Streptococcus pneumoniae: Virulence factors and variation. Clin. Microbiol. Infect..

[143] Crosby H.A., Tiwari N., Kwiecinski J.M., Xu Z., Dykstra A., Jenul C., Fuentes E.J., Horswill A.R. (2020). The *Staphylococcus aureus* ArlRS two-component system regulates virulence factor expression through MgrA. Mol. Microbiol..

[144] Mustafa A., Zulfiqar U., Mumtaz M.Z., Radziemska M., Haider F.U., Holatko J., Hammershmiedt T., Naveed M., Ali H., Kintl A. (2023). Nickel (Ni) phytotoxicity and detoxification mechanisms: A review. Chemosphere.

[145] Mora D., Arioli S. (2014). Microbial urease in health and disease. PLoS Pathog..

[146] Chen C., Xu C., Qian D., Yu Q., Huang M., Zhou L., Qin J.G., Chen L., Li E. (2020). Growth and health status of Pacific white shrimp, Litopenaeus vannamei, exposed to chronic water born cobalt. Fish Shellfish. Immunol..

[147] Gopinath K., Venclovas Č., Ioerger T.R., Sacchettini J.C., McKinney J.D., Mizrahi V., Warner D.F. (2013). A vitamin B12 transporter in Mycobacterium tuberculosis. Open Biol..

[148] Duell B.L., Su Y.C., Riesbeck K. (2016). Host–pathogen interactions of nontypeable Haemophilus influenzae: From commensal to pathogen. FEBS Lett..

[149] Schwarz G., Mendel R.R., Ribbe M.W. (2009). Molybdenum cofactors, enzymes and pathways. Nature.

[150] Mayer F.L., Wilson D., Hube B. (2013). Candida albicans pathogenicity mechanisms. Virulence.

[151] Jeong G.-J., Khan F., Khan S., Tabassum N., Mehta S., Kim Y.-M. (2023). Pseudomonas aeruginosa virulence attenuation by inhibiting siderophore functions. Appl. Microbiol. Biotechnol..

[152] Ladomersky E., Petris M.J. (2015). Copper tolerance and virulence in bacteria. Metallomics.

[153] Ogunniyi A.D., Mahdi L.K., Jennings M.P., McEwan A.G., McDevitt C.A., Van der Hoek M.B., Bagley C.J., Hoffmann P., Gould K.A., Paton J.C. (2010). Central role of manganese in regulation of stress responses, physiology, and metabolism in Streptococcus pneumoniae. J. Bacteriol..

[154] Kadioglu A., Weiser J.N., Paton J.C., Andrew P.W. (2008). The role of Streptococcus pneumoniae virulence factors in host respiratory colonization and disease. Nat. Rev. Microbiol..

[155] Li M., Yu J., Guo G., Shen H. (2023). Interactions between macrophages and biofilm during Staphylococcus aureus-associated implant infection: Difficulties and solutions. J. Innate Immun..

[156] Michel A., Agerer F., Hauck C.R., Herrmann M., Ullrich J., Hacker J., Ohlsen K. (2006). Global regulatory impact of ClpP protease of Staphylococcus aureus on regulons involved in virulence, oxidative stress response, autolysis, and DNA repair. J. Bacteriol..

[157] Collins C.M., D’Orazio S.E. (1993). Bacterial ureases: Structure, regulation of expression and role in pathogenesis. Mol. Microbiol..

[158] Ansari S., Yamaoka Y. (2017). Survival of Helicobacter pylori in gastric acidic territory. Helicobacter.

[159] Liu J., Li X., Zhu Y., Ge R. (2023). Molecular Mechanisms of Bismuth-containing Drugs Against Helicobacter pylori: A Further Update. Curr. Pharmacol. Rep..

[160] Schalk I.J., Cunrath O. (2016). An overview of the biological metal uptake pathways in P seudomonas aeruginosa. Environ. Microbiol..

[161] Ghssein G., Ezzeddine Z. (2022). A review of Pseudomonas aeruginosa metallophores: Pyoverdine, pyochelin and pseudopaline. Biology.

[162] Kılıç N.K., Stensballe A., Otzen D.E., Dönmez G. (2010). Proteomic changes in response to chromium (VI) toxicity in Pseudomonas aeruginosa. Bioresour. Technol..

[163] Okamoto S., Eltis L.D. (2011). The biological occurrence and trafficking of cobalt. Metallomics.

[164] Salam L.B. (2020). Unravelling the antibiotic and heavy metal resistome of a chronically polluted soil. 3 Biotech.

[165] Westman J., Plumb J., Licht A., Yang M., Allert S., Naglik J.R., Hube B., Grinstein S., Maxson M.E. (2022). Calcium-dependent ESCRT recruitment and lysosome exocytosis maintain epithelial integrity during Candida albicans invasion. Cell Rep..

[166] Jayaseelan S., Ramaswamy D., Dharmaraj S. (2014). Pyocyanin: Production, applications, challenges and new insights. World J. Microbiol. Biotechnol..

[167] Hall S., McDermott C., Anoopkumar-Dukie S., McFarland A.J., Forbes A., Perkins A.V., Davey A.K., Chess-Williams R., Kiefel M.J., Arora D. (2016). Cellular effects of pyocyanin, a secreted virulence factor of Pseudomonas aeruginosa. Toxins.

[168] Norcross E.W., Sanders M.E., Moore III Q.C., Marquart M.E. (2011). Pathogenesis of a clinical ocular strain of Streptococcus pneumoniae and the interaction of pneumolysin with corneal cells. J. Bacteriol. Parasitol..

[169] Schmalstig A.A., Benoit S.L., Misra S.K., Sharp J.S., Maier R.J. (2018). Noncatalytic antioxidant role for Helicobacter pylori urease. J. Bacteriol..

[170] Garland M.M. (2021). Chemical Tools to Interrogate and Inhibit Bacterial Exotoxins.

[171] Losada L., DebRoy C., Radune D., Kim M., Sanka R., Brinkac L., Kariyawasam S., Shelton D., Fratamico P.M., Kapur V. (2016). Whole genome sequencing of diverse Shiga toxin-producing and non-producing Escherichia coli strains reveals a variety of virulence and novel antibiotic resistance plasmids. Plasmid.

[172] Ko Y.-P., Kuipers A., Freitag C.M., Jongerius I., Medina E., van Rooijen W.J., Spaan A.N., van Kessel K.P., Höök M., Rooijakkers S.H. (2013). Phagocytosis escape by a Staphylococcus aureus protein that connects complement and coagulation proteins at the bacterial surface. PLoS Pathog..

[173] Haan L.D., Hirst T.R. (2004). Cholera toxin: A paradigm for multi-functional engagement of cellular mechanisms. Mol. Membr. Biol..

[174] Porcheron G., Garénaux A., Proulx J., Sabri M., Dozois C.M. (2013). Iron, copper, zinc, and manganese transport and regulation in pathogenic Enterobacteria: Correlations between strains, site of infection and the relative importance of the different metal transport systems for virulence. Front. Cell. Infect. Microbiol..

[175] Iatsenko I., Marra A., Boquete J.-P., Peña J., Lemaitre B. (2020). Iron sequestration by transferrin 1 mediates nutritional immunity in Drosophila melanogaster. Proc. Natl. Acad. Sci. USA.

[176] Gurung K., Wertheim B., Falcao Salles J. (2019). The microbiome of pest insects: It is not just bacteria. Entomol. Exp. Appl..

[177] Loulou A., Mastore M., Caramella S., Bhat A.H., Brivio M.F., Machado R.A., Kallel S. (2023). Entomopathogenic potential of bacteria associated with soil-borne nematodes and insect immune responses to their infection. PLoS ONE.

[178] Wojda I. (2017). Immunity of the greater wax moth Galleria mellonella. Insect Sci..

[179] Zhang L.-j., Gallo R.L. (2016). Antimicrobial peptides. Curr. Biol..

[180] Bastakoti S. (2023). Role of zinc in management of plant diseases: A review. Cogent Food Agric..

[181] Glavinic U., Stankovic B., Draskovic V., Stevanovic J., Petrovic T., Lakic N., Stanimirovic Z. (2017). Dietary amino acid and vitamin complex protects honey bee from immunosuppression caused by Nosema ceranae. PLoS ONE.

[182] Shafeeq S., Yesilkaya H., Kloosterman T.G., Narayanan G., Wandel M., Andrew P.W., Kuipers O.P., Morrissey J.A. (2011). The cop operon is required for copper homeostasis and contributes to virulence in Streptococcus pneumoniae. Mol. Microbiol..

[183] Palmer L.D., Skaar E.P. (2016). Transition metals and virulence in bacteria. Annu. Rev. Genet..

[184] Price S.L., Vadyvaloo V., DeMarco J.K., Brady A., Gray P.A., Kehl-Fie T.E., Garneau-Tsodikova S., Perry R.D., Lawrenz M.B. (2021). Yersiniabactin contributes to overcoming zinc restriction during Yersinia pestis infection of mammalian and insect hosts. Proc. Natl. Acad. Sci. USA.

[185] Geiser D.L., Winzerling J.J. (2012). Insect transferrins: Multifunctional proteins. Biochim. Et Biophys. Acta (BBA)-Gen. Subj..

[186] Khan I.U., Qi S.-S., Gul F., Manan S., Rono J.K., Naz M., Shi X.-N., Zhang H., Dai Z.-C., Du D.-L. (2023). A Green Approach Used for Heavy Metals ‘Phytoremediation’Via Invasive Plant Species to Mitigate Environmental Pollution: A Review. Plants.

[187] Sandy M., Butler A. (2009). Microbial iron acquisition: Marine and terrestrial siderophores. Chem. Rev..

[188] Crawford A., Wilson D. (2015). Essential metals at the host–pathogen interface: Nutritional immunity and micronutrient assimilation by human fungal pathogens. FEMS Yeast Res..

[189] Lewis J.P. (2010). Metal uptake in host-pathogen interactions: Role of iron in Porphyromonas gingivalis interactions with host organisms. Periodontol. 2000.

[190] Scandalios J. (2005). Oxidative stress: Molecular perception and transduction of signals triggering antioxidant gene defenses. Braz. J. Med. Biol. Res..

[191] Vasil M.L. (2007). How we learnt about iron acquisition in Pseudomonas aeruginosa: A series of very fortunate events. Biometals.

[192] Roth M. (2023). Oxidative Stress Response: Lessons from the Gram-Negative Bacterium Escherichia coli.

[193] Waterman S.R., Hacham M., Hu G., Zhu X., Park Y.-D., Shin S., Panepinto J., Valyi-Nagy T., Beam C., Husain S. (2007). Role of a CUF1/CTR4 copper regulatory axis in the virulence of Cryptococcus neoformans. J. Clin. Investig..

[194] Pokharel P., Dhakal S., Dozois C.M. (2023). The Diversity of Escherichia coli Pathotypes and Vaccination Strategies against This Versatile Bacterial Pathogen. Microorganisms.

[195] Zhao C., Jia X., Pan Y., Liao S., Zhang S., Ji C., Kuang G., Wu X., Liu Q., Tang Y. (2023). Thioredoxin A of Streptococcus suis Serotype 2 Contributes to Virulence by Inhibiting the Expression of Pentraxin 3 to Promote Survival Within Macrophages. J. Microbiol..

[196] Heindl J.E., Hibbing M.E., Xu J., Natarajan R., Buechlein A.M., Fuqua C. (2016). Discrete responses to limitation for iron and manganese in Agrobacterium tumefaciens: Influence on attachment and biofilm formation. J. Bacteriol..

[197] Fan H.-H., Fang S.-B., Chang Y.-C., Huang S.-T., Huang C.-H., Chang P.-R., Chang W.-C., Yang L.T.-L., Lin P.-C., Cheng H.-Y. (2022). Effects of colonization-associated gene yqiC on global transcriptome, cellular respiration, and oxidative stress in Salmonella typhimurium. J. Biomed. Sci..

[198] Ohradanova-Repic A., Praženicová R., Gebetsberger L., Moskalets T., Skrabana R., Cehlar O., Tajti G., Stockinger H., Leksa V. (2023). Time to Kill and Time to Heal: The Multifaceted Role of Lactoferrin and Lactoferricin in Host Defense. Pharmaceutics.

[199] Marshall N.C., Finlay B.B. (2014). Targeting the type III secretion system to treat bacterial infections. Expert Opin. Ther. Targets.

[200] Koul A., Herget T., Klebl B., Ullrich A. (2004). Interplay between mycobacteria and host signalling pathways. Nat. Rev. Microbiol..

[201] Dobrindt U., Hacker J. (2010). Uropathogens and virulence factors. Urogenit. Infect..

[202] Ding C., Festa R.A., Sun T.S., Wang Z.Y. (2014). Iron and copper as virulence modulators in human fungal pathogens. Mol. Microbiol..

[203] Neyrolles O., Wolschendorf F., Mitra A., Niederweis M. (2015). Mycobacteria, metals, and the macrophage. Immunol. Rev..

[204] Maurya R., Namdeo M. (2021). Superoxide dismutase: A key enzyme for the survival of intracellular pathogens in host. Reactive Oxygen Species.

[205] Bombaywala S., Purohit H.J., Dafale N.A. (2021). Mobility of antibiotic resistance and its co-occurrence with metal resistance in pathogens under oxidative stress. J. Environ. Manag..

[206] Brooks L.R., Mias G.I. (2018). Streptococcus pneumoniae’s virulence and host immunity: Aging, diagnostics, and prevention. Front. Immunol..

[207] Calderone R.A., Fonzi W.A. (2001). Virulence factors of Candida albicans. Trends Microbiol..

[208] Kong C., Neoh H.-M., Nathan S. (2016). Targeting Staphylococcus aureus toxins: A potential form of anti-virulence therapy. Toxins.

[209] Mendel R.R., Bittner F. (2006). Cell biology of molybdenum. Biochim. Biophys. Acta (BBA)-Mol. Cell Res..

[210] Mashburn L.M., Jett A.M., Akins D.R., Whiteley M. (2005). Staphylococcus aureus serves as an iron source for Pseudomonas aeruginosa during in vivo coculture. J. Bacteriol..

[211] Khan A., Singh P., Srivastava A. (2018). Synthesis, nature and utility of universal iron chelator–Siderophore: A review. Microbiol. Res..

[212] Porcheron G., Dozois C.M. (2015). Interplay between iron homeostasis and virulence: Fur and RyhB as major regulators of bacterial pathogenicity. Vet. Microbiol..

[213] Nairz M., Schroll A., Sonnweber T., Weiss G. (2010). The struggle for iron–a metal at the host–pathogen interface. Cell. Microbiol..

[214] Kell D.B., Heyden E.L., Pretorius E. (2020). The biology of lactoferrin, an iron-binding protein that can help defend against viruses and bacteria. Front. Immunol..

[215] Yang X., Peng W., Wang Y., Yan K., Liu Z., Gao T., Yang K., Liu W., Guo R., Li C. (2023). Mutations in troABCD against Copper Overload in a copA Mutant of Streptococcus suis. Appl. Environ. Microbiol..

[216] Focarelli F., Giachino A., Waldron K.J. (2022). Copper microenvironments in the human body define patterns of copper adaptation in pathogenic bacteria. PLoS Pathog..

[217] Virieux-Petit M., Hammer-Dedet F., Aujoulat F., Jumas-Bilak E., Romano-Bertrand S. (2022). From copper tolerance to resistance in pseudomonas aeruginosa towards patho-adaptation and hospital success. Genes.

[218] Secli V., Di Biagio C., Martini A., Michetti E., Pacello F., Ammendola S., Battistoni A. (2023). Localized Infections with P. aeruginosa Strains Defective in Zinc Uptake Reveal That Zebrafish Embryos Recapitulate Nutritional Immunity Responses of Higher Eukaryotes. Int. J. Mol. Sci..

[219] Gabbianelli R., Scotti R., Ammendola S., Petrarca P., Nicolini L., Battistoni A. (2011). Role of ZnuABC and ZinT in Escherichia coliO157: H7 zinc acquisition and interaction with epithelial cells. BMC Microbiol..

[220] Carter E.L., Flugga N., Boer J.L., Mulrooney S.B., Hausinger R.P. (2009). Interplay of metal ions and urease. Metallomics.

[221] Acheson D.W., Luccioli S. (2004). Mucosal immune responses. Best Pract. Res. Clin. Gastroenterol..

[222] Sugimoto S., Iwamoto T., Takada K., Okuda K.-i., Tajima A., Iwase T., Mizunoe Y. (2013). Staphylococcus epidermidis Esp degrades specific proteins associated with Staphylococcus aureus biofilm formation and host-pathogen interaction. J. Bacteriol..

[223] Chhipa H., Joshi P. (2016). Nanofertilisers, nanopesticides and nanosensors in agriculture. Nanosci. Food Agric. 1.

[224] Gauthier G.M., Keller N.P. (2013). Crossover fungal pathogens: The biology and pathogenesis of fungi capable of crossing kingdoms to infect plants and humans. Fungal Genet. Biol..

[225] Vignesh K.S., Deepe G.S. (2016). Immunological orchestration of zinc homeostasis: The battle between host mechanisms and pathogen defenses. Arch. Biochem. Biophys..

[226] Rohr J.R., Barrett C.B., Civitello D.J., Craft M.E., Delius B., DeLeo G.A., Hudson P.J., Jouanard N., Nguyen K.H., Ostfeld R.S. (2019). Emerging human infectious diseases and the links to global food production. Nat. Sustain..

[227] Chassy B.M. (2010). Food safety risks and consumer health. New Biotechnol..

[228] Oerke E.-C. (2006). Crop losses to pests. J. Agric. Sci..

[229] Frei A., Verderosa A.D., Elliott A.G., Zuegg J., Blaskovich M.A. (2023). Metals to combat antimicrobial resistance. Nat. Rev. Chem..

[230] Sekhon B.S. (2014). Nanotechnology in agri-food production: An overview. Nanotechnol. Sci. Appl..

[231] Rai M., Ingle A. (2012). Role of nanotechnology in agriculture with special reference to management of insect pests. Appl. Microbiol. Biotechnol..

[232] Morkunas I., Woźniak A., Mai V.C., Rucińska-Sobkowiak R., Jeandet P. (2018). The role of heavy metals in plant response to biotic stress. Molecules.

[233] Jaber L.R., Ownley B.H. (2018). Can we use entomopathogenic fungi as endophytes for dual biological control of insect pests and plant pathogens?. Biol. Control.

[234] Karman S.B., Diah S.Z.M., Gebeshuber I.C. (2015). Raw materials synthesis from heavy metal industry effluents with bioremediation and phytomining: A biomimetic resource management approach. Adv. Mater. Sci. Eng..

